# Supplementary material for: What is the recovery rate and risk of long-term consequences following a diagnosis of COVID-19? A harmonised, global longitudinal observational study protocol
Source: BMJ Open. 2021 Mar 10;11(3):e043887. doi: 10.1136/bmjopen-2020-043887 (PMC7948153; doi:10.1136/bmjopen-2020-043887)
Supplement: Author's manuscript [file bmjopen-2020-043887.draft_revisions.pdf]

BMJ Open is committed to open peer review. As part of this commitment we make the peer review history of every article we publish publicly available.

When an article is published we post the peer reviewers' comments and the authors' responses online. We also post the versions of the paper that were used during peer review. These are the versions that the peer review comments apply to.

The versions of the paper that follow are the versions that were submitted during the peer review process. They are not the versions of record or the final published versions. They should not be cited or distributed as the published version of this manuscript.

BMJ Open is an open access journal and the full, final, typeset and author-corrected version of record of the manuscript is available on our site with no access controls, subscription charges or pay-per-view fees (http://bmjopen.bmj.com).

If you have any questions on BMJ Open's open peer review process please email info.bmjopen@bmj.com

BMJ Open

What is the recovery rate and risk of long-term consequences following a diagnosis of COVID-19? - A harmonised, global longitudinal observational study

Journal:	*BMJ Open*
Manuscript ID	bmjopen-2020-043887
Article Type:	Protocol
Date Submitted by the Author:	21-Aug-2020

Complete List of Authors:	Sigfrid, Louise; University of Oxford, ISARIC Global Support Centre, Centre for Tropical Medicine and Global Heatlh<br>Cevik, Muge; University of St Andrews, Infection and Global Health Division, School of Medicine<br>Jesudason, Edwin; NHS Lothian, Rehabilitation Medicine<br>Lim, Wei Shen; Nottingham University Hospitals NHS Trust, Respiratory Medicine<br>Rello, Jordi; Hospital Universitari Vall d'Hebron, Centro de Investigación Biomédica en Red – Enfermedades Respiratorias (CIBERES); Université Nîmes-Montpellier, Research Department, CHU Nîmes<br>Amuasi, John; Kwame Nkrumah University of Science and Technology and Kumasi Center for Collaborative Research in Tropical Medicine, School of Public Health<br>Bozza, Fernando; Fundação Oswaldo Cruz<br>Palmieri, Carlo; University of Liverpool, Department of Molecular and Clinical Cancer Medicine, Institute of Systems, Molecular and Integrative Biology; The Clatterbridge Cancer Centre NHS Foundation Trust<br>Munblit, Daniel; I M Sechenov First Moscow State Medical University, Paediatrics; Imperial College London Faculty of Medicine, IInflammation, Repair and Development Section, National Heart and Lung Institute<br>Holter, Jan; Oslo University Hospital, Department of Microbiology; University of Oslo, Institute of Clinical Medicine<br>Kildal, Anders; University Hospital of North Norway, Department of Anesthesiology and Intensive Care<br>Russell, Clark; The University of Edinburgh Centre for Inflammation Research<br>Ho, Antonia; University of Glasgow<br>Turtle, Lance; University of Liverpool, NIHR Health Protection Research Unit in emerging and zoonotic infections, Institute of Infection, Veterinary and Ecological Sciences; Liverpool University Hospitals NHS Foundation Trust, Tropical and Infectious Disease Unit<br>Drake, Thomas ; University of Edinburgh, Centre for Medical Informatics<br>Beltrame, Anna; IRCCS Sacro Cuore Don Calabria Hospital, Department of Infectious Diseases, Tropical and Microbiology<br>Hann, Katrina; Sustainable Health Systems<br>Bangura, Ibrahim; Dorothy Springer Trust<br>Fowler, Robert; Sunnybrook Research Institute, Sunnybrook Health Sciences Institute<br>Lakoh, Sulaiman; Sustainable Health Systems

Berry, Colin; University of Glasgow, Institute of Cardiovascular and Medical Sciences, BHF Glasgow Cardiovascular Research Centre
Lowe, David; Queen Elizabeth University Hospital, Emergency Department
McPeake, Joanne; NHS Greater Glasgow and Clyde; University of Glasgow, Institute of Health and Wellbeing
Hashmi, Madiha; Ziauddin University, Critical Care Medicine; Pakistan Registry of Intensive CarE (PRICE)
Dyrhol-Riise, Anne Margarita; Oslo University Hospital, Dep. of Infectious Diseases, Institute of Clinical Medicine
Donohue, Chloe; University of Liverpool, National Institute of Health Research (NIHR) Health Protection research Unit in Emerging and Zoonotic Infections; University of Liverpool, Institute of Infection and Global Health, Faculty of Health and Life Sciences
Plotkin, Daniel; University of Oxford, ISARIC Global Support Centre, Centre for Tropical Medicine and Global Heatlh
Hardwick, Hayley; University of Liverpool, National Institute of Health Research (NIHR) Health Protection research Unit in Emerging and Zoonotic Infections; University of Liverpool, Institute of Infection and Global Health, Faculty of Health and Life Sciences
Elkheir, Natalie; London School of Hygiene & Tropical Medicine
Lone, Nazir; University of Edinburgh, Usher Institute
Docherty, Annemarie; University of Edinburgh, Centre for Medical Informatics, The Usher Institute
Harrison, Ewen ; University of Edinburgh, Director Centre for Medical Informatics, Usher Institute
Baille, Kenneth; Roslin Institute, Division of Genetics and Genomics
Carson, Gail; University of Oxford, ISARIC Global Support Centre, Centre for Tropical Medicine and Global Health
Semple, Malcolm G; University of Liverpool, Health Protection Research Unit In Emerging and Zoonotic Infections, Institute of Infection, Veterinary and Ecological Sciences; Alder Hey Children's NHS Foundation Trust, University of Liverpool
Scott, Janet; University of Glasgow Centre for Virus Research, MRC

Keywords: COVID-19, INFECTIOUS DISEASES, Public health < INFECTIOUS DISEASES

SCHOLARONE™
Manuscripts

I, the Submitting Author has the right to grant and does grant on behalf of all authors of the Work (as defined in the below author licence), an exclusive licence and/or a non-exclusive licence for contributions from authors who are: i) UK Crown employees; ii) where BMJ has agreed a CC-BY licence shall apply, and/or iii) in accordance with the terms applicable for US Federal Government officers or employees acting as part of their official duties; on a worldwide, perpetual, irrevocable, royalty-free basis to BMJ Publishing Group Ltd ("BMJ") its licensees and where the relevant Journal is co-owned by BMJ to the co-owners of the Journal, to publish the Work in this journal and any other BMJ products and to exploit all rights, as set out in our licence.

The Submitting Author accepts and understands that any supply made under these terms is made by BMJ to the Submitting Author unless you are acting as an employee on behalf of your employer or a postgraduate student of an affiliated institution which is paying any applicable article publishing charge ("APC") for Open Access articles. Where the Submitting Author wishes to make the Work available on an Open Access basis (and intends to pay the relevant APC), the terms of reuse of such Open Access shall be governed by a Creative Commons licence – details of these licences and which Creative Commons licence will apply to this Work are set out in our licence referred to above.

Other than as permitted in any relevant BMJ Author's Self Archiving Policies, I confirm this Work has not been accepted for publication elsewhere, is not being considered for publication elsewhere and does not duplicate material already published. I confirm all authors consent to publication of this Work and authorise the granting of this licence.

For peer review only

What is the recovery rate and risk of long-term consequences following a diagnosis of COVID-19? - A harmonised, global longitudinal observational study

Authors: L. Sigfrid*, M. Cevik*, E. Jesudason, W.S Lim, J. Rello, J. H. Amuasi, F. Bozza, C.Palmieri, D. Munblit, J.C.Holter, A.B. Kildal, L.F. Reyes, C.D. Russell, A.Ho, L.Turtle, T.M. Drake, A. Beltrame, K. Hann, I.R. Bangura, R. Fowler, S. Lakoh, C. Berry, D.J. Lowe, J. McPeake, M. Hashmi, A.M. Dyrhol-Riise, C.Donohue, D.R. Plotkin, H. Hardwick, N. Elkheir, N. Lone, A.B. Docherty, E.M. Harrison, J.K. Baille, G. Carson, M.G. Semple, J.T. Scott

*joint first authors

Affiliations:

Louise Sigfrid, PhD Clinical Research Fellow, Public Health Specialist, ISARIC GSC, Centre for Tropical Medicine and Global Health, University of Oxford, Oxford, UK https://orcid.org/0000-0003-2764-1177

Muge Cevik, Infection and Global Health Division, School of Medicine, University of St Andrews, St Andrews, UK https://orcid.org/0000-0003-1133-3874

Edwin Jesudason, Consultant in Rehabilitation Medicine, NHS Lothian, Edinburgh, United Kingdom

Wei Shen Lim, Department of Respiratory Medicine, Nottingham University Hospitals NHS Trust, Nottingham, UK https://orcid.org/0000-0002-7694-3051

Jordi Rello, Prof. 1. Centro de Investigación Biomédica en Red – Enfermedades Respiratorias (CIBERES), Vall d'Hebron Institute of Research (VHIR), Barcelona, Spain. 2. Research Department, CHU Nîmes, Université Nîmes-Montpellier, Nîmes, France. https://orcid.org/0000-0003-0766-6210

John Humphrey Amuasi (MBChB. MPH. MS. PhD.), School of Public Health, Kwame Nkrumah University of Science and Technology and Kumasi Center for Collaborative Research in Tropical Medicine, Ghana https://orcid.org/0000-0002-8640-2662

Fernando Bozza, Fundação Oswaldo Cruz: Rio de Janeiro, RJ, Brasil https://orcid.org/0000-0003-4878-0256

Carlo Palmieri, MD 1. Department of Molecular and Clinical Cancer Medicine, Institute of Systems, Molecular and Integrative Biology, University of Liverpool, UK; 2. The Clatterbridge Cancer Centre NHS Foundation Trust, Liverpool, UK http://orcid.org/0000-0001-9496-2718

Daniel Munblit, Associate Prof. 1. Department of Paediatrics and Paediatric Infectious Diseases, Institute of Child's Health, Sechenov First Moscow State Medical University (Sechenov University), Moscow, Russia. 2. Inflammation, Repair and Development Section, National Heart and Lung Institute, Faculty of Medicine, Imperial College London, London, United Kingdom https://orcid.org/0000-0001-9652-6856

For peer review only - http://bmjopen.bmj.com/site/about/guidelines.xhtml

For peer review only

Jan Cato Holter, MD, PhD. Specialist in infectious diseases and medical microbiology, 1. Department of Microbiology, Oslo University Hospital, Oslo. 2. Institute of Clinical Medicine, University of Oslo, Oslo. https://orcid.org/0000-0003-1618-5022

Anders Benjamin Kildal, MD, PhD, Specialist in Anesthesiology, Department of Anesthesiology and Intensive Care, University Hospital of North Norway, Tromsø, Norway https://orcid.org/0000-0002-1319-6511

Luis Felipe Reyes, MD, PhD, Universidad de La Sabana, Chía, Colombia, https://orcid.org/0000-0003-1172-6539

Clark D. Russell, Clinical lecturer in Infectious Diseases, Centre for Inflammation Research, University of Edinburgh, UK https://orcid.org/0000-0002-9873-8243

Antonia Ho, Clinical Senior Lecturer/Consultant in Infectious Diseases, University of Glasgow, Glasgow, UK https://orcid.org/0000-0003-1465-3785

Lance Turtle, 1. Senior clinical lecturer in infectious diseases, NIHR Health Protection Research Unit in emerging and zoonotic infections, Institute of Infection, Veterinary and Ecological Sciences, University of Liverpool. 2. Consultant in infectious diseases, Tropical and Infectious Disease Unit, Liverpool University Hospitals NHS Foundation Trust. https://orcid.org/0000-0002-0778-1693

Thomas M Drake, Clinical Research Fellow, Centre for Medical Informatics, University of Edinburgh, Edinburgh, United Kingdom, https://orcid.org/0000-0002-9334-6494

Anna Beltrame, Clinician, Department of Infectious Diseases, Tropical and Microbiology, IRCCS Sacro Cuore Don Calabria Hospital: Negrar di Valpolicella, Italy https://orcid.org/0000-0002-7499-5843

Katrina Hann, Sustainable Health Systems, Freetown, Sierra Leone https://orcid.org/0000-0002-4913-6270

Ibrahim Richard Bangura, Data Manager, Dorothy Springer Trust, Sierra Leone

Rob Fowler, Department of Medicine and Department of Critical Care Medicine, Sunnybrook Hospital, University of Toronto. Toronto, Canada

Sulaiman Lakoh, Sustainable Health Systems, Freetown, Sierra Leone https://orcid.org/0000-0002-7639-0004

Colin Berry, Prof. Institute of Cardiovascular and Medical Sciences, BHF Glasgow Cardiovascular Research Centre, University of Glasgow, Glasgow, UK, https://orcid.org/0000-0002-4547-8636

David J Lowe, Emergency Department, Queen Elizabeth University Hospital, Glasgow, Scotland, UK https://orcid.org/0000-0003-4866-2049

Joanne McPeake, 1. NHS Greater Glasgow and Clyde 2. University of Glasgow Institute of Health and Wellbeing 3. THIS Institute, University of Cambridge ORCID: https://orcid.org/0000-0001-8206-6801

Madiha Hashmi, 1. Critical Care Medicine, Ziauddin University, Karachi, Pakistan 2. Pakistan Registry of Intensive

For peer review only

CarE (PRICE), Pakistan. 3. South East Asian Research in Critical care Health (SEARCH).

Dyrhol-Riise Anne Margarita, MD, Prof. Dep. of Infectious Diseases, Oslo University hospital, Institute of Clinical Medicine, University of Oslo, Norway. https://orcid.org/0000-0003-4009-4032

Chloe Donohue, 1. National Institute of Health Research (NIHR) Health Protection research Unit in Emerging and Zoonotic Infections, Liverpool, UK. 2. Institute of Infection and Global Health, Faculty of Health and Life Sciences, University of Liverpool, Liverpool, UK

Daniel R Plotkin, ISARIC Global Support Centre, Centre for Tropical Medicine and Global Health, Nuffield Department of Medicine, University of Oxford, Oxford, UK https://orcid.org/0000-0001-9454-1382

Hayley Hardwick, 1. National Institute of Health Research (NIHR) Health Protection research Unit in Emerging and Zoonotic Infections, Liverpool, UK. 2. Institute of Infection and Global Health, Faculty of Health and Life Sciences, University of Liverpool, Liverpool, UK

https://orcid.org/0000-0003-3421-6435

Natalie Elkheir, Clinical research fellow, London School of Hygiene and Tropical Medicine, London, UK

https://orcid.org/0000-0002-1786-8530

Nazir Lone, Usher Institute, Edinburgh, UK https://orcid.org/0000-0003-2707-2779

Annemarie B. Docherty, 1. Centre for Medical Informatics, Usher Institute, University of Edinburgh, Edinburgh, UK. 2. [2]Intensive Care Unit, Royal Infirmary Edinburgh, Edinburgh, UK

Ewen M Harrison, Prof. Director Centre for Medical Informatics, Usher Institute, University of Edinburgh, Edinburgh, UK https://orcid.org/0000-0002-5018-3066

Kenneth J Baillie, Roslin Institute, University of Edinburgh, Edinburgh, UK ORCID:0000-0001-5258-793X

Gail Carson, ISARIC, Centre for Tropical Medicine and Global Health, Nuffield Department of Medicine, University of Oxford, Oxford, UK https://orcid.org/0000-0001-8439-9933

Malcolm G. Semple. Health Protection Research Unit In Emerging and Zoonotic Infections, Institute of Infection, Veterinary and Ecological Sciences, University of Liverpool, UK 2. Respiratory Medicine, Alder Hey Children's NHS Foundation Trust, Eaton Road, Liverpool, UK

https://orcid.org/0000-0001-9700-0418

Janet T Scott, MRC-University of Glasgow Centre for Virus Research, Glasgow, UK

https://orcid.org/0000-0001-8030-5223

Corresponding author:

Louise Sigfrid, ISARIC Global Support Centre, NDM, Centre for Tropical Medicine and Global Health, University of Oxford, Oxford, UK

E-mail: louise.sigfrid@gmail.com

For peer review only - http://bmjopen.bmj.com/site/about/guidelines.xhtml

Abstract:

Introduction

Very little is known about possible clinical sequelae that may persist after resolution of the acute Coronavirus Disease 2019 (COVID-19). A recent longitudinal cohort from Italy including 143 patients recovered after hospitalisation with COVID-19 reported that 87% had at least one ongoing symptom at 60 day follow-up. Early indications suggest that patients with COVID-19 may need even more psychological support than typical ICU patients. The assessment of risk factors for longer term consequences requires a longitudinal study linked to data on pre-existing conditions and care received during the acute phase of illness. We invite hospitals and healthcare centres globally to collaborate and take part in the study.

Methods and analysis

This is an international open-access prospective, observational multi-site study. It will enrol patients following a diagnosis of COVID-19. Tier 1 is developed for following up patients day 28 post-discharge, additionally at 3 to 6 months intervals. This module can be used to identify sub-sets of patients experiencing specific symptomatology or syndromes for further follow up. A Tier 2 module will be developed for in-clinic, in-depth follow up. The primary aim is to characterise physical consequences in patients post-COVID-19. Secondary aim includes estimating the frequency of and risk factors for post-COVID- 19 medical sequalae, psychosocial consequences and post-COVID-19 mortality. A subset of patients will have sampling to characterize longer term antibody, innate and cell-mediated immune responses to SARS-CoV-2.

Ethics and dissemination

This collaborative, open-access study aims to characterize the frequency of and risk factors for long-term consequences and characterise the immune response over time in patients following a diagnosis of COVID-19 and facilitate standardized and longitudinal data collection globally. The outcomes of this study will inform strategies to prevent long term consequences; inform clinical management, direct rehabilitation, and inform public health management to reduce overall morbidity and improve outcomes of COVID-19.

Protocol registration number: osf.io/c5rw3/

Protocol version: 3 August 2020

EuroQol ID: 37035

Key words: COVID-19, post-COVID sequelae, long-term consequences, SARS-CoV-2, observational study

For peer review only - http://bmjopen.bmj.com/site/about/guidelines.xhtml

protocol

Article summary

Strengths and limitations of this study

- As an international prospective, observational study we provide open-access standardised tools that can be adapted by any site interested in following up patients with COVID-19, for independent or combined analysis, to forward knowledge into short and long term consequences of COVID-19.
- This study aims to inform strategies to prevent longer term sequalae; inform clinical management, rehabilitation, and public health management strategies to reduce morbidity and improve outcomes.
- The protocol will be used for a sub-set of patients, already included in the existing cohort of more than 85,973 individuals hospitalized with confirmed COVID-19 infection across 42 countries (as of 20 July 2020), using the ISARIC/WHO standardized Core- or RAPID Case Report Forms (CRFs).
- The data will be linked with data on pre-existing comorbidities, presentation, clinical care and treatments documented in the existing cohort already documented using the ISARIC/WHO standardized Core- or RAPID CRFs.
- The data collection tool is developed to facilitate wide dissemination and uptake, by enabling patient self-assessment, however, follow up of patients requires consent and resources, which might limit the uptake and bias the data towards countries /sites with capacity to follow up patients over time.

For peer review only

For peer review only - http://bmjopen.bmj.com/site/about/guidelines.xhtml

Introduction

Coronavirus Disease 2019 (COVID-19), caused by Severe Acute Respiratory Syndrome Coronavirus-2 (SARS-CoV-2) infection, can lead to a diverse range of clinical manifestations, ranging from an asymptomatic infection to an acute respiratory distress syndrome, and multiorgan failure with high risk of mortality (1). It is established that SARS-CoV-2 not only infects the respiratory tract but that ensuing viral replication and immune response may also affect other organs, which can lead to a risk of heart, renal and liver injury, in addition to an acute systemic inflammatory response and accompanying circulatory shock (2-4). While most people have uncomplicated recoveries, some have prolonged illness even after recovery from the acute illness (5-8). Identifying longer-term potential consequences and relationship with the acute illness is important for the management of patients, in particular, understanding how these interact and affect those already living with other conditions such as cardiovascular disease and cancer will be paramount.

However, very little is known about possible clinical sequelae that may persist after the resolution of acute infection. A recent longitudinal cohort of 143 patients followed after hospitalisation from COVID-19 in Italy, reported that 87% had at least one ongoing symptom, most (55%) with 3 or more symptoms at 60 day follow up, fatigue (53%), dyspnoea (43%), joint pain (27%) and chest pain (22%) being the most common. COVID-19 was associated with worsened quality of life among 44% of patients. (6) Prolonged course of illness has also been reported among people with mild COVID-19 who did not require hospitalisation (5, 8, 9).

Increasing evidence also suggests that infection with Sars-CoV-2 can cause neurological consequences, (2) including altered mental status, comprising encephalopathy or encephalitis and primary psychiatric diagnoses (10). While these symptoms arise acutely during the course of infection, less is known about the possible long-term consequences. Severely affected COVID-19 cases experience high levels of proinflammatory cytokines and acute respiratory dysfunction which often require assisted ventilation. These are known factors suggested to cause cognitive decline (2, 11).

Post-traumatic stress disorder (PTSD) and other consequences after intensive care unit (ICU) stay has been well documented previously (12, 13). A systematic review of consequences after hospitalisation or ICU stay for severe acute respiratory infection (SARS-CoV) and Middle East respiratory syndrome coronavirus (MERS-CoV) found consequences up to 6 months after discharge. Common consequences besides impaired diffusing

For peer review only - http://bmjopen.bmj.com/site/about/guidelines.xhtml

capacity for carbon monoxide and reduced exercise capacity were PTSD (39%), depression (33%) and anxiety (30%) (14).

The UK government predicted in March that 45% of patients will need some form of medical or social input after recovery from COVID-19 and that 4% will require more focused, ongoing intense rehabilitation in a bedded setting (7). Early indications suggest patients with COVID-19 will need even more psychological support than typical post-ICU patients because of higher levels of “survivors’ guilt” and PTSD (15). In addition, the characteristics of the initial cellular immune and antibody response to SARS-CoV-2 have not been fully defined and it is not known if the immune responses generated by infection provides long-term protective immunity. Identifying multidisciplinary consequences through high quality, global studies throughout the course of COVID-19 is important for the acute and longer-term management of patients (4, 16).

The emerging data and anecdotal evidence of long-term recovery and persistent debilitating symptoms highlight the need for robust, standardised studies to assess the risk of and risk factors for sequalae after COVID -19. The purpose of this study is to establish a longitudinal cohort of patients with COVID -19 post-discharge, to characterize risk of long-term consequences and immune responses over time in different populations globally. This will inform strategies to prevent sequalae; inform clinical management, rehabilitation, and public health management strategies to reduce morbidity and improve outcomes.

Methods and analysis

This protocol has been developed by the International Severe Acute Respiratory and emerging Infection Consortium (ISARIC) COVID-19 follow up working group, and informed by a wide range of global stakeholders with expertise in clinical research, outbreak research, infectious disease, epidemiology, respiratory, critical care, rehabilitation, neurology, psychology, rheumatology, cardiology, oncology and public health medicine.

Study design

This is an international prospective, observational multi-site study to assess risk of and risk factors for longer term physical and psychosocial consequences of COVID-19 and immune response over time.

For peer review only - http://bmjopen.bmj.com/site/about/guidelines.xhtml

Population and setting

This protocol builds on the ISARIC/WHO COVID-19 clinical characterization protocol and tools already in operation. The initial follow up module (Tier 1) will be used for a sub-set of patients at participating sites, already included in the existing cohort of more than 85,973 individuals hospitalized with confirmed COVID-19 infection across 42 countries (as of 20 July 2020), using the ISARIC/WHO standardized Core- or RAPID Case Report Forms (CRFs) (17). These CRFs collect data on demographics, pre-existing comorbidities and risk factors, signs and symptoms experienced during the acute phase, and care and treatments received during hospitalization. This data will be linked to the follow up data. The Core- and RAPID CRFs can be completed retrospectively for patients not yet included. New clinical sites are invited to take part in this global collaborative effort and use these open-access tools for independent or combined studies.

Specific inclusion and exclusion criteria are as follows:

Inclusion criteria

- People aged 16 years and older
- Laboratory or physician confirmed COVID -19
- 28 days (-0/+3 months) after discharge from hospital or health centre
- Person (or family member/carer for patients who lack capacity) consent to participate

Inclusion of vulnerable participants

The case report form and validated tools are developed for young people (>16 years of age) and adults, including pregnant women, but not validated for children. A separate case report form will be developed for children.

Serial follow up

The follow up modules are designed in a tiered approach to be adapted depending on local resources and research needs. The Tier 1 case report form is designed to enable patient self-assessment to facilitated distribution to all patients that fit the inclusion criteria. It is developed for following up patients day 28 post-discharge, additionally at 3 to 6 months interval, with or without sampling for as many years as required (Figure 1). By being designed for patient self-completion to be administered via an online link or as a paper form, it allows wide distribution at low resource need. It can also be completed via in-clinic or telephone assessments during check-ups or for patients that are still hospitalised. The module will collect data on demographics, hospital

For peer review only - http://bmjopen.bmj.com/site/about/guidelines.xhtml

stay and re-admissions, all-cause and cause specific mortality (after the initial index event), specific consequences including; deep vein thrombosis (DVT), pulmonary embolism, recent febrile illness, new persistent symptoms, quality of life (measured by EQ-5D-5L), dyspnoea (assessed using MRC dyspnoea scale), difficulties in functioning (UN/Washington disability score), lifestyle and socioeconomic data. The ISARIC collaborative follow up study is registered with EuroQol. Sites who wish to adopt the survey for independent studies, outside of the ISARIC collaboration need to register to use EQ-5D-5L tool with EuroQol.

Figure 1. Schematic overview of the follow up data and sample time-frames

Sub-studies

The Tier 1 follow up module can be used to identify sub-sets of patients experiencing specific symptomatology or syndromes for further follow up. The Tier 2 follow up module will be developed for in-clinic, in-depth follow up. By using a tiered approach, additional specialist modules can be added for more complex follow up of emerging consequences in a flexible, adaptable way (Figure 2).

Biological samples

The CRF is to be used on its own for data collection or in a sub-set of patients in combination with sampling (respiratory samples, serum, blood, stool, urine), for immunology, pathophysiology and other studies (16). It builds on the ISARIC/WHO Clinical Characterization Protocol (CCP) (18, 19). The CCP is designed for any severe or potentially severe acute infection of public health interest, such as COVID-19 (19). It is a standardized protocol for data and biological samples to be collected rapidly in a globally harmonised manner. (20) The CCP can be used for the rapid, coordinated clinical investigation of confirmed cases of COVID-19. It is designed in a tiered approach to be adapted depending on resources and includes different level of sampling schedules (acute phase and follow up) that can be adapted depending on resources, to be combined with patient data collection using the acute phase CRFs and the follow up CRF.

Outcomes

The primary outcome of this study is to characterise physical and psychosocial consequences in patients post-COVID- 19 infection. Secondary outcomes include estimating the risk of and risk factors for post-COVID- 19 medical sequalae, psychosocial consequences and post-COVID-19 mortality. A subset of patients will have

For peer review only - http://bmjopen.bmj.com/site/about/guidelines.xhtml

For peer review only

sampling to characterize longer term antibody, innate and cell-mediated immune responses to SARS-CoV-2 and to evaluate risk of and risk factors for re-infection with SARS-CoV-2 during re-exposure.

Figure 2. ISARIC's adaptable Covid-19 Follow-Up Protocol framework

Data collection and entry

A standardized COVID-19 follow up CRF was developed through a series of virtual working group meetings and e-mail iterations. The CRF was piloted on patients in four settings in three countries and feedback incorporated into the final form (21). The CRF is designed for patient self-assessment, via online link or paper form, or to be completed during in-clinic or telephone follow-up appointment. The CRF is available open access on the ISARIC website (21) and as an electronic CRF (eCRF) on the ISARIC hosted database. The eCRF can be distributed directly to patients via an online link.

Statistical analysis plan

Using the data, we will test for differences in outcomes across important demographic groups (age categories, sex, ethnicity, socioeconomic deprivation, comorbidities), specific exposures (severe COVID-19, critical care admission, ventilation) and initial clinical sequelae (complications on their index admission for COVID-19). We plan to use this platform to conduct timely analyses which coincide with public health or scientific need. Given these requirements, new questions we have not specified within this protocol may arise. Where this occurs, we will develop analysis plans prior to undertaking analyses, which will be made available on request. The data collected through the follow up module will be linked with data on demographics, comorbidities, clinical characteristics, care and treatments collected using the ISARIC/WHO Core- or RAPID COVID-19 CRF (15). The plan below presents our guiding statistical framework.

Entered data will be summarised first by using simple probabilistic statistics. Categorical data will be explored using frequencies and percentages, with differences in disease severity and treatment groups tested for using Chi-square tests or fisher's exact test where cell counts are under five. For continuous data, distribution will be established using histograms and density plots. Data that are normally distributed will be summarised using group mean averages and standard deviation as a measure of central tendency. For non-parametric data, the median average will be used and presented alongside 25th and 75th centiles. Differences in normally distributed continuous data will be tested using Welch's two sample t-tests for 2 group data and ANOVA for three or more

For peer review only - http://bmjopen.bmj.com/site/about/guidelines.xhtml

groups. Mann-Whitney U will be used to compare differences across two groups or Kruskall-Wallis tests for three or more groups, where data follow a non-parametric distribution.

Outcomes will be expressed in three ways; 1) binary event data (for presence or absence of outcome of interest), 2) change over time (for continuous or ordinal data) or as 3) time to event data (for patients with serial measurements). We will calculate changes over time across symptom and outcome variables and use these changes over time to compare the effect of treatments or exposures on these outcomes. Time to event data will be captured for those who complete serial assessment forms. These data will be presented as Kaplan-Meier plots and differences tested for using log-rank tests. Competing risks (including death) will be accounted for using censoring.

To identify which patients are likely to develop persistent complications, functional impairment or reduced quality of life, we will use multilevel models to adjust for potential confounders. Level I fixed effects will include patient-level explanatory variables (i.e. age, sex) and level II or III random effects will include site and country in the case where research questions require differences in country to be accounted for. Explanatory variables will be entered into models on the basis of clinical plausibility and final model selection guided by maximisation of the adjusted R^2 value and minimisation of the Akaike information criterion (AIC) or Bayesian information criterion (BIC). For binary event data, multilevel logistic regression will be used, and estimates presented as odds ratios alongside the corresponding 95% confidence interval. For continuous data, linear or generalised linear regression will be used, and estimates presented as model coefficients, with 95% confidence intervals. Finally, time to event data will be presented as survival probability or hazard ratios, with 95% confidence intervals.

Statistical significance will be taken at the level of $P < 0.05$ *a-priori*. Analyses will be conducted in secure R (R Foundation for Statistical Computing, Vienna, AUT) or STATA (StataCorp LLC, TX, USA) environments.

Data sharing

Sites who wish to utilise ISARIC's COVID-19 data management and hosting support can email ncov@isaric.org to gain access to a secure data capture and management system. All systems are free to use and supported by ISARIC data management specialists. Sites who submit data to ISARIC will sign a Terms of Submission, enabling use of the data in collaborative analysis by ISARIC partners.

For peer review only - http://bmjopen.bmj.com/site/about/guidelines.xhtml

Patient and public involvement

The survey has been developed with a wide range of professionals and been piloted with patients in different settings in three different countries. The feedback from the pilot has been incorporated into the final version of the Tier 1 survey. Since follow-up modules will involve both phone/online methods and will also include a subset who will come in-person, we aim to consult local community-based groups and organizations on the best approaches to achieving high levels of participation and limiting the attrition rate.

Ethics and dissemination

Since the emergence of SARS-CoV-2 in December 2019, there has been a considerable global effort to characterise the virus and clinical course of disease. This has included identification of the virus, rapid design and development of diagnostics, host cell receptor identification, and insights into early epidemiological and clinical parameters. However, very little is known about possible clinical sequelae that may persist after resolution of the acute COVID-19. Emerging data indicates impact of COVID-19 infection on not only the respiratory system but also, the kidneys, liver, neurological, psychological and multisystem inflammatory syndrome (2, 4, 10, 22). Moreover, with increasing reports of long-term consequences, there is an urgent need to characterise the risk of short and chronic consequences and risk factors and biomarkers for patients at risk of sequelae, to inform preventative and rehabilitation strategies.

The assessment of risk factors for longer term consequences requires a longitudinal study linked to data on pre-existing conditions and patient data and care received during the acute phase. A subset of patients included in the ISARIC/WHO clinical characterisation protocol has provided consent to be contacted for follow up. Additionally, sites taking part in the global follow up study will apply for consent to follow up patients confirmed with COVID-19, following the local ethical committee procedures. These standardised protocols and tools will serve as a standardised template that can be modified as appropriate and needed at each site. With this study we aim to characterize the risk of long-term consequences and nature of innate and adaptive immune responses over time in patients following a diagnosis of COVID-19. We will collect data on wide range of outcomes including hospital stay and re-admissions, all-cause and cause specific mortality (after the initial index event), consequences e.g. DVT, pulmonary embolism, recent febrile illness, new, persistent symptoms, EQ-5D-5L, MRC dyspnoea scale, UN/Washington disability score, lifestyle and employment data. Data from combined analysis will be disseminated through the ISARIC website and in open-access publications, under group authorship.

For peer review only - http://bmjopen.bmj.com/site/about/guidelines.xhtml

The follow up module is developed as an open-access, flexible tool to be adopted and adapted as appropriate depending on need and resources by any site globally interested in following up patients with COVID-19 over time, to facilitate standardized data collection globally and combined analysis. The outcomes of this study will inform strategies to prevent risk of consequences; clinical management, rehabilitation, and public health management, to reduce morbidity and improve outcomes. By standardising data collection, and providing open-access, adaptable tools, focused on key data variables, it can optimize data quality and reduce the burden of research on staff, while collecting the most relevant information to inform clinical care guidelines and public health. The tools will be made available in a range of languages.

Data statement

ISARIC stands by the principles of data sharing in public health emergencies. ISARIC supported studies will share quality data in a timely, valid, and governed manner to inform public health policy and benefit patient care. The ISARIC-hosted data platform enables rapid and harmonised operationalisation of data collection to a secure database (23). Ownership and control of the data entered are retained by those who enter the data. Sites can contribute data to combined analysis upon permission. Technical appendix, statistical code and datasets are available from https://isaric.tghn.org/ or by contacting: ncov@isaric.org. Research outcomes will be disseminated via the ISARIC website and published in peer-reviewed scientific journals, under a collaborative group authorship. The data contributors will share the outcome results with key stakeholders to inform public health response, policy development and implementation. To efficiently make findings from this project visible and accessible to a wide range of stakeholders as well as to networks of individuals and institutions, we will utilise social media, including the ISARIC and partner institution's websites. The consortium will manage a page dedicated to this project, that will incorporate all the tools available. The webpage will provide not only updates on the progress of the project and component activities and events, but also information on research.

This follow up study is developed as an open-access tool to be adopted as appropriate by any site interested in following up patients with COVID-19 over time, to facilitate standardized data collection globally to enable combined analysis. New sites globally are invited to join the study at any time. The outcomes of this study will inform strategies to prevent risk of consequences; clinical management, rehabilitation and public health management needs to reduce morbidity and improve outcomes. We invite hospitals and healthcare centres globally to collaborate and take part in the study.

For peer review only - http://bmjopen.bmj.com/site/about/guidelines.xhtml

Acknowledgments

We would like to thank the ISARIC global clinical characterisation group, ISARIC4C and all the clinicians, nurses and researchers contributing COVID-19 clinical patient data and samples, which will be linked with the follow up data and all the patients that have consented to be followed up. We would like to acknowledge WHO, whose working groups contributed to development of the CCP and associated tools. Moreover, Sarah Moore, Romans Matulevics, Peter Bannister, Katherine Maskell, Liliana Resende and Anneli Sandström for administrative, graphic design and dissemination support.

Author contributions: JTS and LS lead on the development of the follow up protocol and tools in collaboration with members of the ISARIC COVID-19 follow up working group (JR, JHA, FB, CP, DM, JCH, ABK, A-MDR, KH, IRB, AB, MH, RF, SL, LFP, AH, HH, CD, NE, LT, CR, MC) and external specialists (EJ, WSL, DJL, CB, JM, NL). TD, LS, JTS, ABD, EH developed the statistical analysis plan. DP managed the database set up. AH, LT, ABK, JR, CP piloted the CRF. MGS, JKB, EH, ABD, GC provided scientific advice. LS and MC lead on drafting of the protocol and manuscript, with contributions from all co-authors. All authors reviewed and approved the final manuscript.

Word count: 2983

Competing interest statement: No competing interests declared.

Ethical approval: Ethical approval has been given by the Universidad de La Sabana's IRB (US-MED-504), Colombia; Ghana Health Service Ethics Review Committee and at th Comitato Etico di Brescia, Italy, South-Eastern Norway Regional Health Authority, Norway (Ref 106624). In the UK, for day – 28 follow up as part of the UK CCP approved by the South Central - Oxford C Research Ethics Committee in England (Ref 13/SC/0149) and the Scotland A Research Ethics Committee (Ref 20/SS/0028). The protocol is under review at the Comitato Etico per la sperimentazione Clinica delle Province di Verona e Rovigo, Italy, the Sechenov University Ethics Committee, Moscow, Russian Federation and the University of Witwatersrand Human Research Ethics Committee (Medical), Johannesburg, South Africa and is being submitted to the National Ethics Commitee (CONEP), Rio de Janeiro, Brazil, and in Freetown, Sierra Leone.

Funding statement: This work was supported by the Department for International Development and Wellcome [215091/Z/18/Z] and the Bill & Melinda Gates Foundation [OPP1209135]. CP would like to acknowledge the support of the Liverpool Experimental Cancer Medicine Centre (Grant Reference: C18616/A25153) and The Clatterbridge Cancer Centre Charity. CB acknowledges the support from British Heart Foundation RE/18/6134217.

For peer review only - http://bmjopen.bmj.com/site/about/guidelines.xhtml

References

1. Cevik M, Bamford CGG, Ho A. COVID-19 pandemic-a focused review for clinicians. Clin Microbiol Infect. 2020;26(7):842-7.
2. Heneka MT, Golenbock D, Latz E, Morgan D, Brown R. Immediate and long-term consequences of COVID-19 infections for the development of neurological disease. Alzheimer's research & therapy. 2020;12(1):69.
3. Connors J, Levy J. COVID-19 and its implications for thrombosis and anticoagulation. Blood. 2020;135(23):2033-40.
4. Zaim S, Chong JH, Sankaranarayanan V, Harky A. COVID-19 and Multiorgan Response. Current problems in cardiology. 2020;45(8):100618.
5. Rayner C, Lokugamage A, Molokhia M. Covid-19: Prolonged and relapsing course of illness has implications for returning workers BMJ. 2020.
6. Carfì A, Bernabei R, Landi F, Group ftGAC-P-ACS. Persistent Symptoms in Patients After Acute COVID-19. JAMA. 2020.
7. HM Government, NHS. COVID-19 Hospital Discharge Service Requirements UK; 2020 20 March 2020.
8. Tenforde MW, Kim SS, Lindsell CJ, Rose EB, Shapiro NI, Files DC, et al. Symptom Duration and Risk Factors for Delayed Return to Usual Health Among Outpatients with COVID-19 in a Multistate Health Care Systems Network — United States, March–June 2020 2020 [
9. Assaf G, Davis H, McCorkell L, Wei H, O'Neill B, Akrami A, et al. What Does COVID-19 Recovery Actually Look Like? An Analysis of the Prolonged COVID-19 Symptoms Survey by Patient-Led Research Team. London, UK: The COVID-19 Body Politic Slack Group; 2020. Contract No.: 2 May 2020.
10. Varatharaj A, Thomas N, Ellul MA, Davies NWS, Pollak TA, Tenorio EL, et al. Neurological and neuropsychiatric complications of COVID-19 in 153 patients: a UK-wide surveillance study. The lancet Psychiatry. 2020.
11. Sasannejad C, Ely EW, Lahiri S. Long-term cognitive impairment after acute respiratory distress syndrome: a review of clinical impact and pathophysiological mechanisms. Crit Care. 2019;23(1):352-.
12. Parker AM, Sricharoenchai T, Raparla S, Schneck KW, Bienvenu OJ, Needham DM. Posttraumatic stress disorder in critical illness survivors: a metaanalysis. Critical care medicine. 2015;43(5):1121-9.
13. Davydow DS, Gifford JM, Desai SV, Needham DM, Bienvenu OJ. Posttraumatic stress disorder in general intensive care unit survivors: a systematic review. General hospital psychiatry. 2008;30(5):421-34.
14. Ahmed H, Patel K, Greenwood DC, Halpin S, Lewthwaite P, Salawu A, et al. Long-term clinical outcomes in survivors of severe acute respiratory syndrome and Middle East respiratory syndrome coronavirus outbreaks after hospitalisation or ICU admission: A systematic review and meta-analysis. Journal of rehabilitation medicine. 2020;52(5):jrm00063.
15. Thornton J. Covid-19: the challenge of patient rehabilitation after intensive care. BMJ. 2020;369:m1787.
16. Holmes EA, O'Connor RC, Perry VH, Tracey I, Wessely S, Arseneault L, et al. Multidisciplinary research priorities for the COVID-19 pandemic: a call for action for mental health science. The Lancet Psychiatry. 2020;7(6):547-60.
17. ISARIC, WHO. Covid-19 Case Report Form Oxford, UK: ISARIC; 2020 [Available from: https://isaric.tghn.org/covid-19-clinical-research-resources/.
18. ISARIC, WHO. Clinical Characterisation Protocol: ISARIC; 2012 [version 3.2:[Available from: https://isaric.tghn.org/clinical-characterisation-protocol-ccp/.
19. Dunning JW, Merson L, Rohde GGU, Gao Z, Semple MG, Tran D, et al. Open source clinical science for emerging infections. Lancet Infect Dis. 2014;14(1):8-9.
20. Akhvlediani T, Ali SM, Angus DC, Arabi YM, Ashraf S, Baillie JK, et al. Global outbreak research: harmony not hegemony. The Lancet Infectious Diseases. 2020;20(7):770-2.
21. ISARIC. ISARIC Global COVID-19 Long term follow up study Oxford2020 [Available from: https://isaric.tghn.org/CCP/isaric-global-covid-19-long-term-follow-study/.
22. Lew H, Oh-Park M, Cifu D. The War on COVID-19 Pandemic: Role of Rehabilitation Professionals and Hospitals. American journal of physical medicine & rehabilitation. 2020;99(7):571-2.
23. Harris PA, Taylor R, Thielke R, Payne J, Gonzalez N, Conde JG. Research electronic data capture (REDCap)--a metadata-driven methodology and workflow process for providing translational research informatics support. Journal of biomedical informatics. 2009;42(2):377-81.

For peer review only - http://bmjopen.bmj.com/site/about/guidelines.xhtml

Figure 1. Schematic overview of the follow up data and sample timeframes

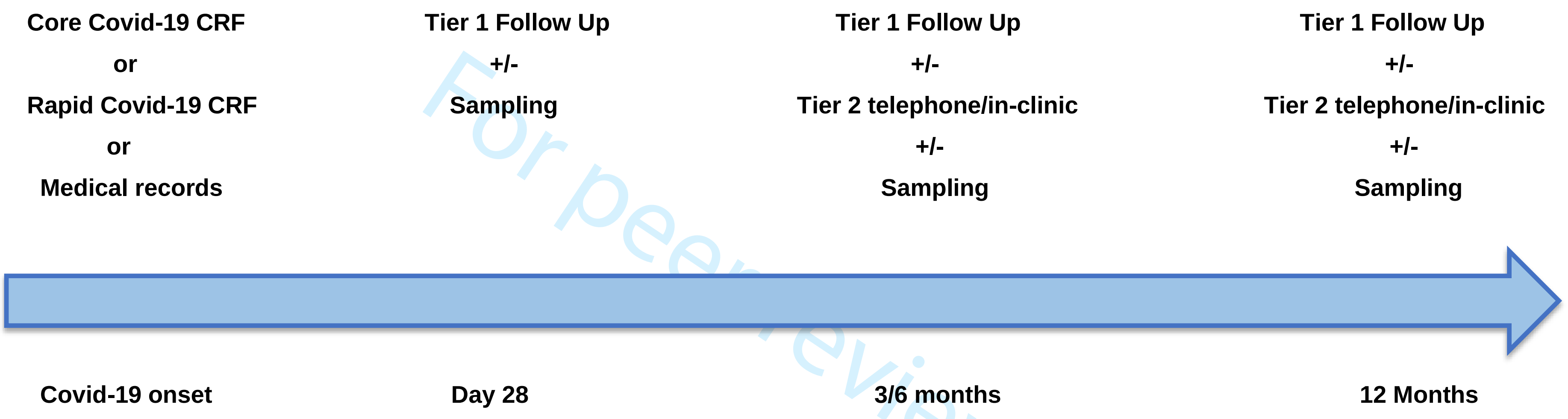

Abbreviations: CRF: Case Report Form

For peer review only

For peer review only - http://bmjopen.bmj.com/site/about/guidelines.xhtml

Figure 2. ISARIC's adaptable Covid-19 Follow-Up Protocol framework

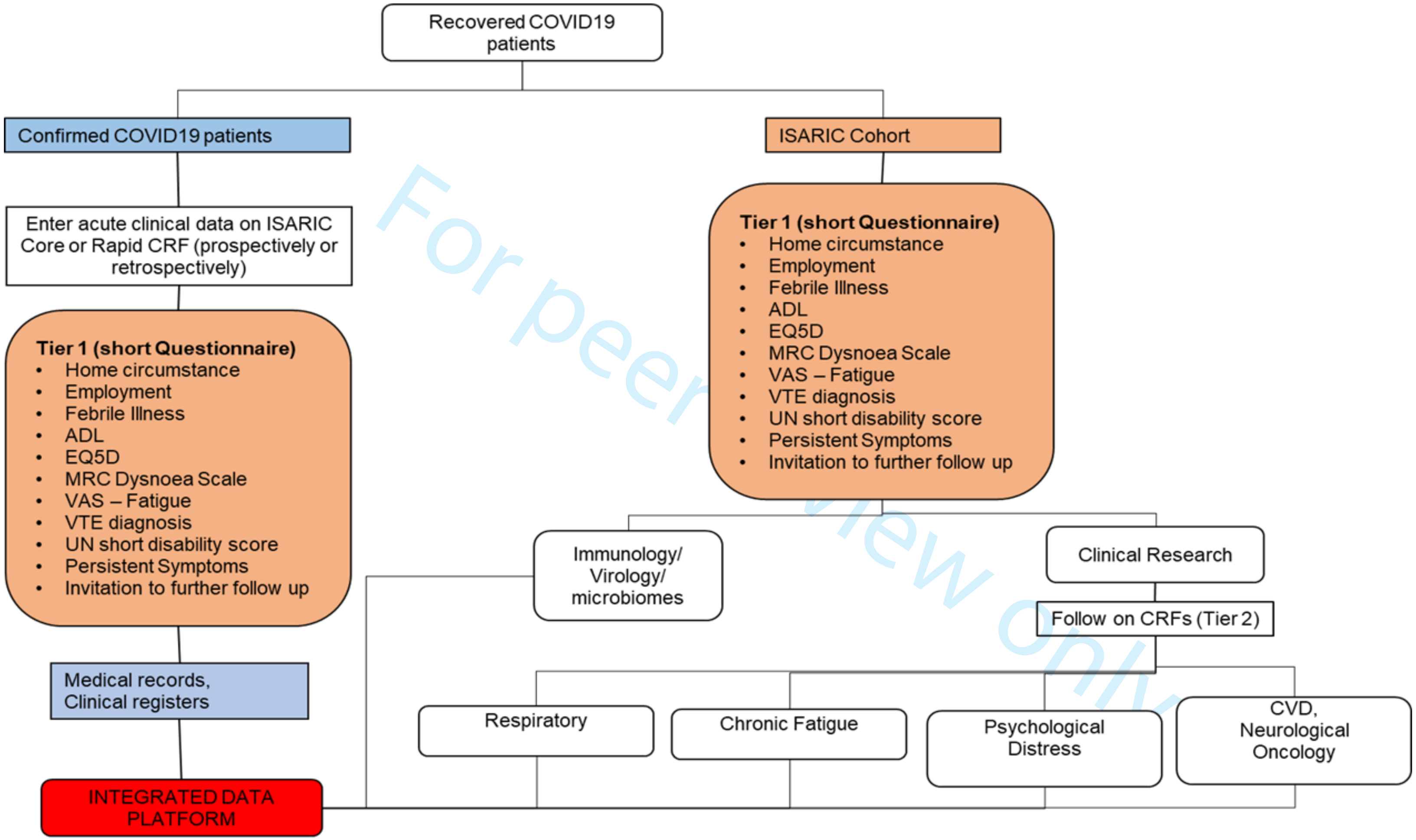

Abbreviations: CRF: Case Report Form, ADL: Activites of daily living, MRC: Medical Research Council, VAS: Visual analogue scale, VTE: Venous thromboembolism, UN: United Nations, ISARIC: International Severe Acute Respiratory and emerging Infection Consortium, CVD: Cardiovascular disease

For peer review only - http://bmjopen.bmj.com/site/about/guidelines.xhtml

BMJ Open

What is the recovery rate and risk of long-term consequences following a diagnosis of COVID-19? – a harmonised, global longitudinal observational study protocol

Journal:	*BMJ Open*
Manuscript ID	bmjopen-2020-043887.R1
Article Type:	Protocol
Date Submitted by the Author:	04-Dec-2020
Complete List of Authors:	Sigfrid, Louise; University of Oxford, ISARIC Global Support Centre, Centre for Tropical Medicine and Global Heatlh<br>Cevik, Muge; University of St Andrews, Infection and Global Health Division, School of Medicine<br>Jesudason, Edwin; NHS Lothian, Rehabilitation Medicine<br>Lim, Wei Shen; Nottingham University Hospitals NHS Trust, Respiratory Medicine<br>Rello, Jordi; Hospital Universitari Vall d'Hebron, Centro de Investigación Biomédica en Red – Enfermedades Respiratorias (CIBERES); Université Nîmes-Montpellier, Research Department, CHU Nîmes<br>Amuasi, John; Kwame Nkrumah University of Science and Technology, School of Public Health<br>Bozza, Fernando; Fundação Oswaldo Cruz<br>Palmieri, Carlo; University of Liverpool, Department of Molecular and Clinical Cancer Medicine, Institute of Systems, Molecular and Integrative Biology; Clatterbridge Cancer Centre NHS Foundation Trust<br>Munblit, Daniel; I M Sechenov First Moscow State Medical University, Paediatrics; Imperial College London Faculty of Medicine, IInflammation, Repair and Development Section, National Heart and Lung Institute<br>Holter, Jan; Oslo University Hospital, Department of Microbiology; University of Oslo, Institute of Clinical Medicine<br>Kildal, Anders Benjamin; University Hospital of North Norway, Department of Anesthesiology and Intensive Care<br>Reyes, Luis; Universidad de La Sabana<br>Russell, Clark; The University of Edinburgh Centre for Inflammation Research<br>Ho, Antonia; University of Glasgow<br>Turtle, Lance; University of Liverpool, NIHR Health Protection Research Unit in emerging and zoonotic infections, Institute of Infection, Veterinary and Ecological Sciences; Liverpool University Hospitals NHS Foundation Trust, Tropical and Infectious Disease Unit<br>Drake, Thomas ; The University of Edinburgh, Centre for Medical Informatics<br>Beltrame, Anna; IRCCS Sacro Cuore Don Calabria Hospital, Department of Infectious Diseases, Tropical and Microbiology<br>Hann, Katrina; Sustainable Health Systems<br>Bangura, Ibrahim; Dorothy Springer Trust<br>Fowler, Robert; Sunnybrook Research Institute, Sunnybrook Health

	Sciences Institute<br>Lakoh, Sulaiman; Sustainable Health Systems<br>Berry, Colin; University of Glasgow, Institute of Cardiovascular and Medical Sciences, BHF Glasgow Cardiovascular Research Centre<br>Lowe, David; Queen Elizabeth University Hospital, Emergency Department<br>McPeake, Joanne; NHS Greater Glasgow and Clyde; University of Glasgow, Institute of Health and Wellbeing<br>Hashmi, Madiha; Ziauddin University, Critical Care Medicine<br>Dyrhol-Riise, Anne Margarita; Oslo University Hospital, Dep. of Infectious Diseases, Institute of Clinical Medicine<br>Donohue, Chloe; University of Liverpool, National Institute of Health Research (NIHR) Health Protection research Unit in Emerging and Zoonotic Infections; University of Liverpool, Institute of Infection and Global Health, Faculty of Health and Life Sciences<br>Plotkin, Daniel; University of Oxford, ISARIC Global Support Centre, Centre for Tropical Medicine and Global Heatlh<br>Hardwick, Hayley; University of Liverpool, National Institute of Health Research (NIHR) Health Protection research Unit in Emerging and Zoonotic Infections; University of Liverpool, Institute of Infection and Global Health, Faculty of Health and Life Sciences<br>Elkheir, Natalie; London School of Hygiene & Tropical Medicine<br>Lone, Nazir; The University of Edinburgh, Usher Institute<br>Docherty, Annemarie; University of Edinburgh, Centre for Medical Informatics, The Usher Institute<br>Harrison, Ewen ; The University of Edinburgh, Director Centre for Medical Informatics, Usher Institute<br>Baille, J; The University of Edinburgh The Roslin Institute, Division of Genetics and Genomics<br>Carson, Gail; University of Oxford, ISARIC Global Support Centre, Centre for Tropical Medicine and Global Health<br>Semple, Malcolm G; University of Liverpool, Health Protection Research Unit In Emerging and Zoonotic Infections, Institute of Infection, Veterinary and Ecological Sciences; Alder Hey Children's NHS Foundation Trust, University of Liverpool<br>Scott, Janet; University of Glasgow Centre for Virus Research, MRC
**Primary Subject Heading**:	Infectious diseases
Secondary Subject Heading:	Global health
Keywords:	COVID-19, INFECTIOUS DISEASES, Public health < INFECTIOUS DISEASES

SCHOLARONE™
Manuscripts

I, the Submitting Author has the right to grant and does grant on behalf of all authors of the Work (as defined in the below author licence), an exclusive licence and/or a non-exclusive licence for contributions from authors who are: i) UK Crown employees; ii) where BMJ has agreed a CC-BY licence shall apply, and/or iii) in accordance with the terms applicable for US Federal Government officers or employees acting as part of their official duties; on a worldwide, perpetual, irrevocable, royalty-free basis to BMJ Publishing Group Ltd ("BMJ") its licensees and where the relevant Journal is co-owned by BMJ to the co-owners of the Journal, to publish the Work in this journal and any other BMJ products and to exploit all rights, as set out in our licence.

The Submitting Author accepts and understands that any supply made under these terms is made by BMJ to the Submitting Author unless you are acting as an employee on behalf of your employer or a postgraduate student of an affiliated institution which is paying any applicable article publishing charge ("APC") for Open Access articles. Where the Submitting Author wishes to make the Work available on an Open Access basis (and intends to pay the relevant APC), the terms of reuse of such Open Access shall be governed by a Creative Commons licence – details of these licences and which Creative Commons licence will apply to this Work are set out in our licence referred to above.

Other than as permitted in any relevant BMJ Author's Self Archiving Policies, I confirm this Work has not been accepted for publication elsewhere, is not being considered for publication elsewhere and does not duplicate material already published. I confirm all authors consent to publication of this Work and authorise the granting of this licence.

For peer review only

What is the recovery rate and risk of long-term consequences following a diagnosis of COVID-19? – a harmonised, global longitudinal observational study protocol

Authors: L. Sigfrid*, M. Cevik*, E. Jesudason, W.S Lim, J. Rello, J. H. Amuasi, F. Bozza, C.Palmieri, D. Munblit, J.C.Holter, A.B. Kildal, L.F. Reyes, C.D. Russell, A.Ho, L.Turtle, T.M. Drake, A. Beltrame, K. Hann, I.R. Bangura, R. Fowler, S. Lakoh, C. Berry, D.J. Lowe, J. McPeake, M. Hashmi, A.M. Dyrhol-Riise, C.Donohue, D.R. Plotkin, H. Hardwick, N. Elkheir, N. Lone, A.B. Docherty, E.M. Harrison, J.K. Baille, G. Carson, M.G. Semple, J.T. Scott

*joint first authors

Affiliations:

Louise Sigfrid, PhD Clinical Research Fellow, Public Health Specialist, ISARIC GSC, Centre for Tropical Medicine and Global Health, University of Oxford, Oxford, UK https://orcid.org/0000-0003-2764-1177

Muge Cevik, Infection and Global Health Division, School of Medicine, University of St Andrews, St Andrews, UK https://orcid.org/0000-0003-1133-3874

Edwin Jesudason, Consultant in Rehabilitation Medicine, NHS Lothian, Edinburgh, United Kingdom

Wei Shen Lim, Department of Respiratory Medicine, Nottingham University Hospitals NHS Trust, Nottingham, UK https://orcid.org/0000-0002-7694-3051

Jordi Rello, Prof. 1. Centro de Investigación Biomédica en Red – Enfermedades Respiratorias (CIBERES), Vall d'Hebron Institute of Research (VHIR), Barcelona, Spain. 2. Research Department, CHU Nîmes, Université Nîmes-Montpellier, Nîmes, France. https://orcid.org/0000-0003-0766-6210

John Humphrey Amuasi (MBChB. MPH. MS. PhD.), School of Public Health, Kwame Nkrumah University of Science and Technology, Ghana https://orcid.org/0000-0002-8640-2662

Fernando Bozza, MD, Fundação Oswaldo Cruz: Rio de Janeiro, RJ, Brasil https://orcid.org/0000-0003-4878-0256

Carlo Palmieri, MD 1. Department of Molecular and Clinical Cancer Medicine, Institute of Systems, Molecular and Integrative Biology, University of Liverpool, UK; 2. The Clatterbridge Cancer Centre NHS Foundation Trust, Liverpool, UK http://orcid.org/0000-0001-9496-2718

Daniel Munblit, Associate Prof. 1. Department of Paediatrics and Paediatric Infectious Diseases, Institute of Child's Health, Sechenov First Moscow State Medical University (Sechenov University), Moscow,

For peer review only

For peer review only - http://bmjopen.bmj.com/site/about/guidelines.xhtml

Russia. 2. Inflammation, Repair and Development Section, National Heart and Lung Institute, Faculty of Medicine, Imperial College London, London, United Kingdom https://orcid.org/0000-0001-9652-6856

Jan Cato Holter, MD, PhD. Specialist in infectious diseases and medical microbiology, 1. Department of Microbiology, Oslo University Hospital, Oslo. 2. Institute of Clinical Medicine, University of Oslo, Oslo. https://orcid.org/0000-0003-1618-5022

Anders Benjamin Kildal, MD, PhD, Specialist in Anesthesiology, Department of Anesthesiology and Intensive Care, University Hospital of North Norway, Tromsø, Norway https://orcid.org/0000-0002-1319-6511

Luis Felipe Reyes, MD, PhD, Universidad de La Sabana, Chía, Colombia, https://orcid.org/0000-0003-1172-6539

Clark D. Russell, MD, Clinical lecturer in Infectious Diseases, Centre for Inflammation Research, University of Edinburgh, UK https://orcid.org/0000-0002-9873-8243

Antonia Ho, MD, PhD, Clinical Senior Lecturer/Consultant in Infectious Diseases, University of Glasgow, Glasgow, UK https://orcid.org/0000-0003-1465-3785

Lance Turtle, MD, PhD, 1. Senior clinical lecturer in infectious diseases, NIHR Health Protection Research Unit in emerging and zoonotic infections, Institute of Infection, Veterinary and Ecological Sciences, University of Liverpool. 2. Consultant in infectious diseases, Tropical and Infectious Disease Unit, Liverpool University Hospitals NHS Foundation Trust. https://orcid.org/0000-0002-0778-1693

Thomas M Drake, MD, PhD, Clinical Research Fellow, Centre for Medical Informatics, University of Edinburgh, Edinburgh, UK, https://orcid.org/0000-0002-9334-6494

Anna Beltrame, MD, Clinician, Department of Infectious Diseases, Tropical and Microbiology, IRCCS Sacro Cuore Don Calabria Hospital: Negrar di Valpolicella, Italy https://orcid.org/0000-0002-7499-5843

Katrina Hann, MD, Sustainable Health Systems, Freetown, Sierra Leone https://orcid.org/0000-0002-4913-6270

Ibrahim Richard Bangura, Data Manager, Dorothy Springer Trust, Sierra Leone

Rob Fowler, MD, PhD. Department of Medicine and Department of Critical Care Medicine, Sunnybrook Hospital, University of Toronto. Toronto, Canada

Sulaiman Lakoh, MD, Sustainable Health Systems, Freetown, Sierra Leone https://orcid.org/0000-0002-7639-0004

For peer review only

Colin Berry, Prof. Institute of Cardiovascular and Medical Sciences, BHF Glasgow Cardiovascular Research Centre, University of Glasgow, Glasgow, UK, https://orcid.org/0000-0002-4547-8636

David J Lowe, MD,Emergency Department, Queen Elizabeth University Hospital, Glasgow, Scotland, UK https://orcid.org/0000-0003-4866-2049

Joanne McPeake, 1. NHS Greater Glasgow and Clyde 2. University of Glasgow Institute of Health and Wellbeing 3. THIS Institute, University of Cambridge ORCID: https://orcid.org/0000-0001-8206-6801

Madiha Hashmi, 1. Critical Care Medicine, Ziauddin University, Karachi, Pakistan 2. Pakistan Registry of Intensive CarE (PRICE), Pakistan. 3. South East Asian Research in Critical care Health (SEARCH).

Dyrhol-Riise Anne Margarita, MD, Prof. Dep. of Infectious Diseases, Oslo University hospital, Institute of Clinical Medicine, University of Oslo, Norway. https://orcid.org/0000-0003-4009-4032

Chloe Donohue, 1. National Institute of Health Research (NIHR) Health Protection research Unit in Emerging and Zoonotic Infections, Liverpool, UK. 2. Institute of Infection and Global Health, Faculty of Health and Life Sciences, University of Liverpool, Liverpool, UK

Daniel R Plotkin, ISARIC Global Support Centre, Centre for Tropical Medicine and Global Health, Nuffield Department of Medicine, University of Oxford, Oxford, UK https://orcid.org/0000-0001-9454-1382

Hayley Hardwick, 1. National Institute of Health Research (NIHR) Health Protection research Unit in Emerging and Zoonotic Infections, Liverpool, UK. 2. Institute of Infection and Global Health, Faculty of Health and Life Sciences, University of Liverpool, Liverpool, UK https://orcid.org/0000-0003-3421-6435

Natalie Elkheir, MD, Clinical research fellow, London School of Hygiene and Tropical Medicine, London, UK https://orcid.org/0000-0002-1786-8530

Nazir Lone, MD, Usher Institute, Edinburgh, UK https://orcid.org/0000-0003-2707-2779

Annemarie B. Docherty, MD, 1. Centre for Medical Informatics, Usher Institute, University of Edinburgh, Edinburgh, UK. 2. [2]Intensive Care Unit, Royal Infirmary Edinburgh, Edinburgh, UK

Ewen M Harrison, Prof. Director Centre for Medical Informatics, Usher Institute, University of Edinburgh, Edinburgh, UK https://orcid.org/0000-0002-5018-3066

J Kenneth Baillie, MD, Roslin Institute, University of Edinburgh, Edinburgh, UK ORCID:0000-0001-5258-793X

Gail Carson, ISARIC, Centre for Tropical Medicine and Global Health, Nuffield Department of Medicine, University of Oxford, Oxford, UK https://orcid.org/0000-0001-8439-9933

For peer review only

For peer review only - http://bmjopen.bmj.com/site/about/guidelines.xhtml

Malcolm G. Semple. Prof. Health Protection Research Unit In Emerging and Zoonotic Infections, Institute of Infection, Veterinary and Ecological Sciences, University of Liverpool, UK 2. Respiratory Medicine, Alder Hey Children's NHS Foundation Trust, Eaton Road, Liverpool, UK

https://orcid.org/0000-0001-9700-0418

Janet T Scott, MD, PhD, MRC-University of Glasgow Centre for Virus Research, Glasgow, UK

https://orcid.org/0000-0001-8030-5223

Corresponding author:

Louise Sigfrid, MD, PhD, ISARIC Global Support Centre, NDM, Centre for Tropical Medicine and Global Health, University of Oxford, Oxford, UK

E-mail: louise.sigfrid@gmail.com

Abstract:

Introduction

Very little is known about possible clinical sequelae that may persist after resolution of the acute Coronavirus Disease 2019 (COVID-19). A recent longitudinal cohort from Italy including 143 patients recovered after hospitalisation with COVID-19 reported that 87% had at least one ongoing symptom at 60-day follow-up. Early indications suggest that patients with COVID-19 may need even more psychological support than typical ICU patients. The assessment of risk factors for longer term consequences requires a longitudinal study linked to data on pre-existing conditions and care received during the acute phase of illness. The primary aim of this study is to characterise physical and psychosocial sequelae in patients post-COVID-19 hospital discharge.

Methods and analysis

This is an international open-access prospective, observational multi-site study. This protocol is linked with the International severe acute respiratory and emerging infection consortium (ISARIC) and the WHO's clinical characterisation protocol, which includes patients with suspected or confirmed COVID-19 during hospitalisation. This protocol will follow up a subset of patients with confirmed COVID-19 using standardised surveys to measure longer term physical and psychosocial sequelae. The data will be linked with the acute phase data. Statistical analyses will be undertaken to characterise groups most

For peer review only - http://bmjopen.bmj.com/site/about/guidelines.xhtml

likely to be affected by sequelae of COVID-19. The open-access follow up survey can be used as a data collection tool by other follow up studies, to facilitate data harmonisation, and to identify sub-sets of patients for further in-depth follow up .

Ethics and dissemination

The protocol and survey are open-access to enable low resourced sites to join the study, to facilitate global standardized, longitudinal data collection. The outcomes of this study will inform strategies to prevent long term consequences; inform clinical management, interventional studies, rehabilitation, and public health management to reduce overall morbidity and improve long term outcomes of COVID-19.

Protocol registration number: osf.io/c5rw3/

Protocol version: 3 August 2020

EuroQol ID: 37035

Key words: COVID-19, post-acute COVID-19 sequelae, long-term consequences, SARS-CoV-2, observational study protocol, open-access, pandemic research

Article summary

Strengths and limitations of this study

- This is an open-access protocol and data collection forms to facilitate standardised, multisite data collection to forward knowledge into long term consequences of COVID-19.
- This study aims to inform strategies to prevent longer term sequalae; inform clinical management, rehabilitation, and public health management strategies to reduce morbidity and improve outcomes.
- The protocol will be used to follow up a sub-set of patients, already included in the existing ISARIC cohort of more than 88, 463 individuals hospitalized with COVID-19 across 42 countries (as of 4 October 2020).
- The follow up data will be linked with acute-phase data already documented using the ISARIC/WHO standardized Core- or RAPID case report forms (CRFs).

For peer review only - http://bmjopen.bmj.com/site/about/guidelines.xhtml

- The data collection tool is developed to facilitate wide dissemination and uptake with limited resources, to mitigate resources limitations during the pandemic.

Introduction

Coronavirus Disease 2019 (COVID-19), caused by Severe Acute Respiratory Syndrome Coronavirus-2 (SARS-CoV-2) infection, can lead to a diverse range of clinical manifestations, ranging from an asymptomatic infection to an acute respiratory distress syndrome, and multiorgan failure with high risk of mortality (1). It is established that SARS-CoV-2 not only infects the respiratory tract but that ensuing viral replication and immune response may also affect other organs, which can lead to a risk of heart, renal and liver injury, in addition to an acute systemic inflammatory response and accompanying circulatory shock (2-4). While most people have uncomplicated recoveries, some have prolonged illness even after recovery from the acute illness (5-7). Identifying longer-term potential consequences and relationship with the acute illness is important for the management of patients, in particular, understanding how these interact and affect those already living with other conditions such as cardiovascular disease and cancer will be paramount.

However, very little is known about possible clinical sequelae that may persist after the resolution of acute infection. A recent longitudinal cohort of 143 patients followed after hospitalisation from COVID-19 in Italy, reported that 87% had at least one ongoing symptom, most (55%) with 3 or more symptoms at 60 day follow up, fatigue (53%), dyspnoea (43%), joint pain (27%) and chest pain (22%) being the most common. COVID-19 was associated with worsened quality of life among 44% of patients. (6) Prolonged course of illness has also been reported among people with mild COVID-19 who did not require hospitalisation (5, 7, 8).

Increasing evidence also suggests that infection with SARS-CoV-2 can cause neurological consequences, (2) including altered mental status, comprising encephalopathy or encephalitis and primary psychiatric diagnoses (9). While these symptoms arise acutely during the course of infection, less is known about the possible long-term consequences. Severely affected COVID-19 cases

For peer review only - http://bmjopen.bmj.com/site/about/guidelines.xhtml

experience high levels of proinflammatory cytokines and acute respiratory dysfunction which often require assisted ventilation. These are known factors suggested to cause cognitive decline (2, 10).

Post-traumatic stress disorder (PTSD) and post-intensive care syndrome after intensive care unit (ICU) stay has been well documented previously (11-13). A systematic review of consequences after hospitalisation or ICU stay for severe acute respiratory infection (SARS-CoV) and Middle East respiratory syndrome coronavirus (MERS-CoV) found sequelae up to 6 months after discharge. Common consequences besides impaired diffusing capacity for carbon monoxide and reduced exercise capacity were PTSD (39%), depression (33%) and anxiety (30%) (14). Additionally, serial CT scans post discharge after SARS-CoV showed a gradual healing of pulmonary injury, with pulmonary consequences lasting more than 6 months post discharge. (15)

Early indications suggest patients with COVID-19 will need even more psychological support than typical post-ICU patients because of higher levels of “survivors’ guilt” and PTSD (16). In addition, the characteristics of the initial cellular immune and antibody response to SARS-CoV-2 have not been fully defined and it is not known if the immune responses generated by infection provides long-term protective immunity. Identifying multidisciplinary sequalae and complications through high quality, global studies throughout the course of COVID-19 is important for the acute and longer-term management of patients (4, 17).

The emerging data and anecdotal evidence of long-term recovery and persistent debilitating symptoms highlight the need for robust, standardised studies to assess the risk of and risk factors for COVID -19 sequalae. The purpose of this study is to establish a longitudinal cohort of patients with COVID -19 post-discharge to characterise the risk of long-term consequences over time in different populations globally. (18) The primary outcome is to characterise physical and psychosocial consequences in patients post-COVID- 19 infection. Secondary outcomes include estimating the risk of and risk factors for post-COVID-19 medical sequalae, psychosocial consequences and long-term outcomes. The results will inform strategies to prevent long term consequences; inform clinical management, interventional research,

For peer review only - http://bmjopen.bmj.com/site/about/guidelines.xhtml

direct rehabilitation, and inform public health management to reduce overall morbidity and improve outcomes of COVID-19.

Methods and analysis

This protocol and data collection surveys have been developed by the ISARIC COVID-19 global follow up working group and informed by a wide range of global stakeholders with expertise in clinical research, outbreak research, infectious disease, epidemiology, respiratory, critical care, rehabilitation, neurology, psychology, rheumatology, cardiology, oncology and public health medicine.(18)

Study design

This is an international prospective, observational multi-site study to assess risk of and risk factors for longer term physical and psychosocial consequences of COVID-19.

The study conforms to research ethics standards and has been approved by research ethics boards in Colombia, Ghana, Italy, Norway and the UK and submitted for ethics approval in additional sites and countries. All sites adopting the protocol are responsible for ensuring that they have local ethical approval in place.

Population and setting

This protocol builds on the ISARIC/WHO COVID-19 clinical characterisation protocol (CCP) and associated data collection forms already in operation, the Core- and Rapid case report forms (CRFs).(19) These CRFs were developed to standardised clinical data collection on patients admitted with suspected or confirmed COVID-19, with clinical data on more than 88,463 individuals hospitalized with confirmed COVID-19 infection across 42 countries documented in a central database (as of 8 Oct. 2020). (20) These CRFs collect data on demographics, pre-existing comorbidities and risk factors, signs and symptoms experienced during the acute phase, and care and treatments received during hospitalization. (19) This acute phase data will be linked with the follow up data to inform analysis. A subset of these patients with confirmed COVID-19, will be followed up over time, documenting data on longer term consequences using the Tier 1 follow up data collection surveys.(18) There is no limit on the number of sites or countries taking part.

For peer review only - http://bmjopen.bmj.com/site/about/guidelines.xhtml

New clinical sites are invited to take part in this global collaborative effort and use these open-access tools. New sites can complete the Core- or RAPID- CRFs prospectively or retrospectively (Figure 1). Sites adopting the protocol and tools for collaborative or independent studies are responsible for ensuring local regulatory and ethical approvals are in place as appropriate.

Specific inclusion and exclusion criteria are as follows:

Inclusion criteria

- People aged 16 years and older
- Laboratory or physician confirmed COVID -19
- At least 1 month post- discharge from hospital or health centre
- Person (or family member/carer for patients who lack capacity) consent to participate

Inclusion of vulnerable participants

The data collection surveys and validated tools are developed for anyone who fit the inclusion criteria, including pregnant women, elderly and those who are immunosuppressed. An aligned study protocol and survey will be developed for following up children.

Outcomes and Procedure

The follow up protocol and survey are designed to be flexible in a tiered approach to be adapted depending on local resources and research needs. The Tier 1 survey is designed to enable patient self-assessment to facilitate distribution to all patients that fit the inclusion criteria, using a range of methods via post, an online link for self-completion or via telephone or in-clinic completion. A combination of methods can be used to reach as a wide population as possible and depending on site resources. The survey can be adapted and adopted as a standardised data collection tool as part of other studies e.g. in combination with sampling and/or diagnostic methods for further analysis (Tier 2) (Figure 1).

Figure 1. ISARIC's adaptable Covid-19 Follow-Up Protocol framework

Abbreviations: ISARIC: International Severe Acute Respiratory and emerging Infection Consortium, MRC: The Medical Research Council, VAS: Visual analogues scale, WHO: World Health Organisation

For peer review only - http://bmjopen.bmj.com/site/about/guidelines.xhtml

Serial follow up

The aim is to follow up patients serially over time, for as long as there is a need and resources at regular intervals. The Tier 1 initial survey will be used at the inital follow up timepoint (at 1 to 4 months post discharge). The Tier 1 ongoing survey will be used at subsequent follow up time points every 3 to 6 months depending on resources (Figure 2). To faciliate combined analysis the time frames in Figure 2 are recommended. These can be adapted depending on local resources and needs. For sites that have capacity, the Tier 1 form can be used to identify people for in clinic follow up or subset of people for serial follow up. By being designed for patient self-completion to be administered via an online link or as a paper form, it allows wide distribution at low resource need. It can also be completed via in-clinic or telephone assessments during check-ups or for patients that are still hospitalised. The module will collect data on demographics, hospital stay and re-admissions, all-cause and cause specific mortality (after the initial index event), specific consequences including; deep vein thrombosis (DVT), pulmonary embolism, recent febrile illness, new or persistent symptoms, quality of life (measured by EQ-5D-5L), dyspnoea (assessed using MRC dyspnoea scale), difficulties in functioning (UN/Washington disability score), lifestyle and socioeconomic data.(18) Medical records will be used to assess if a person is deceased before each follow up time point, and documented in the database. The ISARIC collaborative follow up study is registered with EuroQol. Sites who wish to adopt the survey for independent studies, outside of the ISARIC collaboration need to register to use EQ-5D-5L tool with EuroQol.

Sub-studies

The Tier 1 follow up module can be used to identify sub-sets of patients experiencing specific symptomatology or syndromes for further follow up. By using a tiered approach, additional specialist modules can be added for more complex follow up of emerging consequences in a flexible, adaptable way, of a subset of patients, and combined with sampling and diagnostics (Figure 1 and 2).

Figure 2. Schematic overview of the follow up data time-frames

Serial follow up will continue beyond 12 months at 3 to 6 months‘ interval for up to 3 years depending on resources.

For peer review only - http://bmjopen.bmj.com/site/about/guidelines.xhtml

Abbreviations: ISARIC: International Severe Acute Respiratory and emerging Infection Consortium, WHO: World Health Organisation

Biological samples

The Tier 1 surveys can be used on its own for data collection, or in combination with sampling (e.g. respiratory samples, serum, blood, stool, urine), for immunology, pathophysiology, genomics or other studies (16). This protocol builds on the ISARIC/WHO Clinical Characterisation Protocol (CCP) which includes an adaptable research sample schedule for sites with resources for adding optional research sampling and analysis (19, 21). The CCP is designed for any severe or potentially severe acute infection of public health interest, such as COVID-19. It is a standardized protocol for data and biological samples to be collected rapidly in a globally harmonised manner.(21, 22) The CCP can be used for the rapid, coordinated clinical investigation of confirmed cases of COVID-19. It is also designed in a tiered approach to be adapted depending on resources and includes different levels of sampling schedules (acute phase and follow up) that can be adapted depending on resources, to be combined with patient data collection using the acute phase CRFs and the follow up CRF. (18, 19)

Outcomes

The primary outcome of this study is to characterise physical and psychosocial consequences in patients post-acute COVID- 19. Secondary outcomes include estimating the risk of and risk factors for post-COVID- 19 medical sequalae, psychosocial consequences and post-COVID-19 mortality.

Data collection and entry

A standardised COVID-19 follow up survey aimed for global settings was developed through a series of virtual working group meetings and e-mail iterations. The survey was piloted on patients in four settings in three countries and feedback incorporated into the final form. This Tier 1 follow up survey is designed for patient self-assessment, via online link or paper form, or to be completed during in-clinic or telephone follow-up appointment (Figure 1). (18) The CRF is available open access on the ISARIC website and as an electronic form on the ISARIC hosted REDCap database. (18) The survey can be distributed directly to patients via an online link.

For peer review only - http://bmjopen.bmj.com/site/about/guidelines.xhtml

Statistical analysis plan

Using the data, we will test for differences in outcomes across important demographic groups (age categories, sex, ethnicity, socioeconomic deprivation, comorbidities), specific exposures (severe COVID-19, critical care admission, ventilation) and initial clinical sequelae (complications on their index admission for COVID-19). We plan to use this platform to conduct timely analyses which coincide with public health or scientific need. Given these requirements, new questions we have not specified within this protocol may arise. Where this occurs, we will develop analysis plans prior to undertaking analyses, which will be made available on request. The data collected through the follow up module will be linked with data on demographics, comorbidities, clinical characteristics, care and treatments collected using the ISARIC/WHO Core- or RAPID COVID-19 CRF.(19)

Fields contained within the data collection forms will be combined and if an area of interest is found, the maximal amount of data will be used to investigate this to maintain sample size and power. The plan below presents our guiding statistical framework. Analyses will be developed concurrently with data collection using statistical coding script. At appropriate time intervals, scripts will be run to produce analyses at these timepoints over the course of the project.

As COVID-19 is a new disease, there are no systematically collected long-term data to base formal sample size calculations upon. Therefore, we intend to recruit as many patients as possible. Through the network established already, this is anticipated to be very large. Therefore, as a minimum calculation, to perform logistic regression, we will use at least 10 events for each variable included in the regression. For a regression containing 10 explanatory variables, 100 events would be included within the model for each variable. Assuming a sequelae rate of at least 20%, we would need at least 500 patients which should be adequately achieved. As the field is rapidly evolving, the analyses are likely to change.

Entered data will be summarised first by using simple summary statistics. Categorical data will be explored using frequencies and percentages, with differences in disease severity and treatment groups tested for using Chi-square tests or fisher's exact test where cell counts are under five. For continuous data, distribution will be established using histograms and density plots. Data that are normally

For peer review only - http://bmjopen.bmj.com/site/about/guidelines.xhtml

distributed will be summarised using group mean averages and standard deviation as a measure of central tendency. For non-parametric data, the median average will be used and presented alongside 25th and 75th centiles. Differences in normally distributed continuous data will be tested using Welch's two sample t-tests for 2 group data and ANOVA for three or more groups. Mann-Whitney U will be used to compare differences across two groups or Kruskall-Wallis tests for three or more groups, where data follow a non-parametric distribution.

Outcomes will be expressed in three ways; 1) binary event data (for presence or absence of outcome of interest), 2) change over time (for continuous or ordinal data), or as 3) time to event data (for patients with serial measurements). We will calculate changes over time across symptom and outcome variables and use these changes over time to compare the effect of treatments or exposures on these outcomes. Time to event data will be captured for those who complete serial assessment forms. These data will be presented as Kaplan-Meier plots and differences tested for using log-rank tests. Competing risks (including death) will be accounted for using censoring.

To address the main aim of characterising the middle to long-term impact of COVID-19 on physical and mental health, we will first provide simple summaries of incidence and second, characterise which patients are at risk of developing these. To identify which patients are likely to develop persistent complications, functional impairment or reduced quality of life, we will use multilevel models to adjust for potential confounders. Level I fixed effects will include patient-level explanatory variables (i.e. age, sex) and level II or III random effects will include site and country in the case where research questions require differences in country to be accounted for. Explanatory variables will be entered into models since clinical plausibility and final model selection guided by maximisation of the adjusted R^2 value and minimisation of the Akaike information criterion (AIC) or Bayesian information criterion (BIC). For binary event data, multilevel logistic regression will be used, and estimates presented as odds ratios alongside the corresponding 95% confidence interval. For continuous data, linear or generalised linear regression will be used, and estimates presented as model coefficients, with 95% confidence intervals. Finally, time to event data will be presented as survival probability or hazard ratios, with 95% confidence intervals.

For peer review only - http://bmjopen.bmj.com/site/about/guidelines.xhtml

Statistical significance will be taken at the level of $P < 0.05$ *a-priori*. Analyses will be conducted in secure R (R Foundation for Statistical Computing, Vienna, AUT) or STATA (StataCorp LLC, TX, USA) environments.

Data sharing

Sites who wish to utilise ISARIC's COVID-19 data management and hosting support can email ncov@isaric.org to gain access to a secure data capture and management system. All systems are free to use and supported by ISARIC data management specialists. Sites who submit data to ISARIC will sign a Terms of Submission, enabling use of the data in collaborative analysis by ISARIC partners.

Patient and public involvement

This protocol and the data collection survey has been informed by people living with long Covid, has piloted and provided feedback on the data collection survey, in clinics and via online meetings and e-mail iterations. This included suggestions on the data on symptoms collected and the way questions were asked as well as on the patient information, which were incorporated.

Ethics and dissemination

Since the emergence of SARS-CoV-2 in December 2019, there has been a considerable global effort to characterise the virus and clinical course of disease. However, very little is known about possible clinical sequelae that may persist after resolution of the acute COVID-19 and individual risk factors for prolonged recovery and sequelea. Emerging data indicates impact of COVID-19 infection on not only the respiratory system but also the kidneys, liver, neurological, psychological and multisystem inflammatory syndrome (2, 4, 9, 23). There is an urgent need to characterise the risk of short and chronic sequela and population risk factors for these, to inform preventative and rehabilitation strategies. This study is open access for any sites globally to join the collaboration to characterise COVID-19 in different populations. Providing research tools that can be implemented for free in low resourced settings can increase equity in implementation of and inclusion in clinical research studies.

For peer review only

For peer review only - http://bmjopen.bmj.com/site/about/guidelines.xhtml

The assessment of risk factors for longer term consequences requires a longitudinal study linked to data on pre-existing conditions and patient data and care received during the acute phase. A subset of patients included in the ISARIC/WHO clinical characterisation protocol have provided consent to be contacted for follow up. Additionally, sites taking part in the global follow up study will apply for consent to follow up patients confirmed with COVID-19, following the local ethical committee procedures. These standardised protocols and tools will serve as a standardised template that can be modified as appropriate and needed at each site. With this study we aim to characterise the risk of long-term consequences over time in patients following a diagnosis of COVID-19. We will collect data on a wide range of outcomes including hospital stay and all-cause and cause specific mortality (after the initial index event), new or persistent symptoms and complications, e.g. DVT, pulmonary embolism, recent febrile illness, new, persistent symptoms, EQ-5D-5L, MRC dyspnoea scale, UN/Washington disability score, lifestyle and employment data. Data from combined analysis will be disseminated through the ISARIC website and in open-access publications under group authorship

The follow up module is developed as an open-access, flexible tool to be adopted and adapted as appropriate depending on need and resources by any site globally interested in following up patients with COVID-19 over time, to facilitate standardized data collection globally and combined analysis. The outcomes of this study will inform strategies to prevent risk of consequences; clinical management, rehabilitation, and public health management, to reduce morbidity and improve outcomes. By standardising data collection, and providing open-access, adaptable tools, focused on key data variables, it can optimize data quality and reduce the burden of research on staff, while collecting the most relevant information to inform clinical care guidelines and public health. The Tier 1 follow up survey is available in English, Italian, Portuguese, Russian and Spanish.(18) The survey will be translations into additional languages as required.

Data statement

ISARIC stands by the principles of data sharing in public health emergencies. ISARIC supported studies will share quality data in a timely, valid, and governed manner to inform public health policy and benefit patient care. The ISARIC-hosted data platform enables rapid and harmonised operationalisation of data collection to a secure database (24). Ownership and control of the data entered are retained by those

For peer review only - http://bmjopen.bmj.com/site/about/guidelines.xhtml

who enter the data. Sites can contribute data to combined analysis upon permission. Technical appendix, statistical code and datasets are available from https://isaric.org/document/covid-19-data-management-hosting/ or by contacting: ncov@isaric.org. Research outcomes will be disseminated via the ISARIC website and published in peer-reviewed scientific journals under a collaborative group authorship. The data contributors will share the outcome results with key stakeholders to inform public health response, policy development and implementation. To efficiently make findings from this project visible and accessible to a wide range of stakeholders as well as to networks of individuals and institutions, we will utilise social media, including the ISARIC and partner institution's websites. The consortium will manage a page dedicated to this project that will incorporate all the tools available. The webpage will provide not only updates on the progress of the project and component activities and events, but also information on research.

This follow up study is developed as an open-access tool to be adopted as appropriate by any site interested in following up patients with COVID-19 over time, to facilitate standardised data collection globally to enable combined analysis. New sites globally are invited to join the study at any time. The outcomes of this study will inform strategies to prevent risk of consequences; clinical management, rehabilitation and public health management needs to reduce morbidity and improve outcomes. We invite hospitals and healthcare centres globally to collaborate and take part in the study.

Acknowledgments

We would like to thank the ISARIC global clinical characterisation group, ISARIC4C and all the clinicians, nurses and researchers contributing COVID-19 clinical patient data, which will be linked with the follow up data, and all the patients that have consented to be followed up. We would like to acknowledge WHO, whose working groups contributed to development of the CCP and associated tools. Moreover, Sarah Moore, Romans Matulevics, Laura Merson, Peter Bannister, Katherine Maskell, Eli Harriss and Liliana Resende, Anneli Sandström and Tova Strong for administrative, graphic design and dissemination support.

For peer review only

For peer review only - http://bmjopen.bmj.com/site/about/guidelines.xhtml

Author contributions: JTS and LS lead on the development of the follow up protocol and tools in collaboration with members of the ISARIC COVID-19 follow up working group (JR, JHA, FB, CP, DM, JCH, ABK, A-MDR, KH, IRB, AB, MH, RF, SL, LFR, AH, HH, CD, NE, LT, CR, MC) and external specialists (EJ, WSL, DJL, CB, JM, NL). TD, LFR, LS, JTS, ABD, EH developed the statistical analysis plan. DP managed the database set up. AH, LT, ABK, JR, CP piloted the case report form. MGS, JKB, EH, ABD, GC provided scientific advice. LS and MC lead on drafting of the protocol and manuscript, with contributions from all co-authors. All authors reviewed and approved the final manuscript.

Word count: 3585

Competing interest statement: No competing interests declared.

Ethical approval: Ethical approval has been given by the Universidad de La Sabana's IRB (US-MED-504), Colombia; Ghana Health Service Ethics Review Committee and at the Comitato Etico di Brescia, Italy, South-Eastern Norway Regional Health Authority, Norway (Ref 106624). In the UK, for day – 28 follow up as part of the UK CCP approved by the South Central - Oxford C Research Ethics Committee in England (Ref 13/SC/0149) and the Scotland A Research Ethics Committee (Ref 20/SS/0028). The protocol is under review at the Comitato Etico per la sperimentazione Clinica delle Province di Verona e Rovigo, Italy, the Sechenov University Ethics Committee, Moscow, Russian Federation and the University of Witwatersrand Human Research Ethics Committee (Medical), Johannesburg, South Africa and is being submitted to the National Ethics Committee (CONEP), Rio de Janeiro, Brazil, and in Freetown, Sierra Leone. Sites interested in adopting the protocol as it is or in an adapted version are responsible for ensuring that local sponsorship and ethical approvals in place as appropriate.

Funding statement: This work was supported by the Department for International Development and Wellcome [215091/Z/18/Z] and the Bill & Melinda Gates Foundation [OPP1209135]. CP would like to acknowledge the support of the Liverpool Experimental Cancer Medicine Centre (Grant Reference: C18616/A25153) and The Clatterbridge Cancer Centre Charity.

For peer review only

For peer review only - http://bmjopen.bmj.com/site/about/guidelines.xhtml

For peer review only

For peer review only - http://bmjopen.bmj.com/site/about/guidelines.xhtml

References

1. Cevik M, Bamford CGG, Ho A. COVID-19 pandemic-a focused review for clinicians. Clin Microbiol Infect. 2020;26(7):842-7.
2. Heneka MT, Golenbock D, Latz E, Morgan D, Brown R. Immediate and long-term consequences of COVID-19 infections for the development of neurological disease. Alzheimer's research & therapy. 2020;12(1):69.
3. Connors J, Levy J. COVID-19 and its implications for thrombosis and anticoagulation. Blood. 2020;135(23):2033-40.
4. Zaim S, Chong JH, Sankaranarayanan V, Harky A. COVID-19 and Multiorgan Response. Current problems in cardiology. 2020;45(8):100618.
5. Rayner C, Lokugamage A, Molokhia M. Covid-19: Prolonged and relapsing course of illness has implications for returning workers BMJ. 2020.
6. Carfì A, Bernabei R, Landi F, Group ftGAC-P-ACS. Persistent Symptoms in Patients After Acute COVID-19. JAMA. 2020.
7. Tenforde MW, Kim SS, Lindsell CJ, Rose EB, Shapiro NI, Files DC, et al. Symptom Duration and Risk Factors for Delayed Return to Usual Health Among Outpatients with COVID-19 in a Multistate Health Care Systems Network — United States, March–June 2020 2020 [
8. Assaf G, Davis H, McCorkell L, Wei H, O'Neill B, Akrami A, et al. What Does COVID-19 Recovery Actually Look Like? An Analysis of the Prolonged COVID-19 Symptoms Survey by Patient-Led Research Team. London, UK: The COVID-19 Body Politic Slack Group; 2020. Contract No.: 2 May 2020.
9. Varatharaj A, Thomas N, Ellul MA, Davies NWS, Pollak TA, Tenorio EL, et al. Neurological and neuropsychiatric complications of COVID-19 in 153 patients: a UK-wide surveillance study. The lancet Psychiatry. 2020.
10. Sasannejad C, Ely EW, Lahiri S. Long-term cognitive impairment after acute respiratory distress syndrome: a review of clinical impact and pathophysiological mechanisms. Crit Care. 2019;23(1):352-.
11. Parker AM, Sricharoenchai T, Raparla S, Schneck KW, Bienvenu OJ, Needham DM. Posttraumatic stress disorder in critical illness survivors: a metaanalysis. Critical care medicine. 2015;43(5):1121-9.
12. Davydow DS, Gifford JM, Desai SV, Needham DM, Bienvenu OJ. Posttraumatic stress disorder in general intensive care unit survivors: a systematic review. General hospital psychiatry. 2008;30(5):421-34.
13. Bangash MN, Owen A, Alderman JE, Chotalia M, Patel JM, Parekh D. COVID-19 recovery: potential treatments for post-intensive care syndrome. The Lancet Respiratory Medicine. 2020;8(11):1071-3.
14. Ahmed H, Patel K, Greenwood DC, Halpin S, Lewthwaite P, Salawu A, et al. Long-term clinical outcomes in survivors of severe acute respiratory syndrome and Middle East respiratory syndrome coronavirus outbreaks after hospitalisation or ICU admission: A systematic review and meta-analysis. Journal of rehabilitation medicine. 2020;52(5):jrm00063.
15. Salehi S, Reddy S, Gholamrezanezhad A. Long-term Pulmonary Consequences of Coronavirus Disease 2019 (COVID-19): What We Know and What to Expect. Journal of thoracic imaging. 2020;35(4):W87-w9.
16. Thornton J. Covid-19: the challenge of patient rehabilitation after intensive care. BMJ. 2020;369:m1787.
17. Holmes EA, O'Connor RC, Perry VH, Tracey I, Wessely S, Arseneault L, et al. Multidisciplinary research priorities for the COVID-19 pandemic: a call for action for mental health science. The Lancet Psychiatry. 2020;7(6):547-60.
18. ISARIC. ISARIC Covid-19 long term follow up study Oxford, UK2020 [cited 2020 13 Nov. 2020]. Available from: https://isaric.org/research/covid-19-clinical-research-resources/covid-19-long-term-follow-up-study/.
19. ISARIC. ISARIC/WHO Core- and Rapid- Covid-19 Case Report Forms Oxford, UK2020 [cited 2020 13 Nov.]. Available from: https://isaric.org/research/covid-19-clinical-research-resources/covid-19-crf/.
20. Pritchard M, Dankwa EA, Hall M, Baillie JK, Carson G, Docherty A, et al. ISARIC Clinical Data Report 4 October 2020. medRxiv. 2020:2020.07.17.20155218.

For peer review only - http://bmjopen.bmj.com/site/about/guidelines.xhtml

21. ISARIC, WHO. Clinical Characterisation Protocol (CCP): ISARIC; 2012 [cited 2020 13 Nov.]. version 3.2:[Available from: https://isaric.org/research/covid-19-clinical-research-resources/clinical-characterisation-protocol-ccp/.

22. Akhvlediani T, Ali SM, Angus DC, Arabi YM, Ashraf S, Baillie JK, et al. Global outbreak research: harmony not hegemony. The Lancet Infectious Diseases. 2020;20(7):770-2.

23. Lew H, Oh-Park M, Cifu D. The War on COVID-19 Pandemic: Role of Rehabilitation Professionals and Hospitals. American journal of physical medicine & rehabilitation. 2020;99(7):571-2.

24. Harris PA, Taylor R, Thielke R, Payne J, Gonzalez N, Conde JG. Research electronic data capture (REDCap)--a metadata-driven methodology and workflow process for providing translational research informatics support. Journal of biomedical informatics. 2009;42(2):377-81.

For peer review only

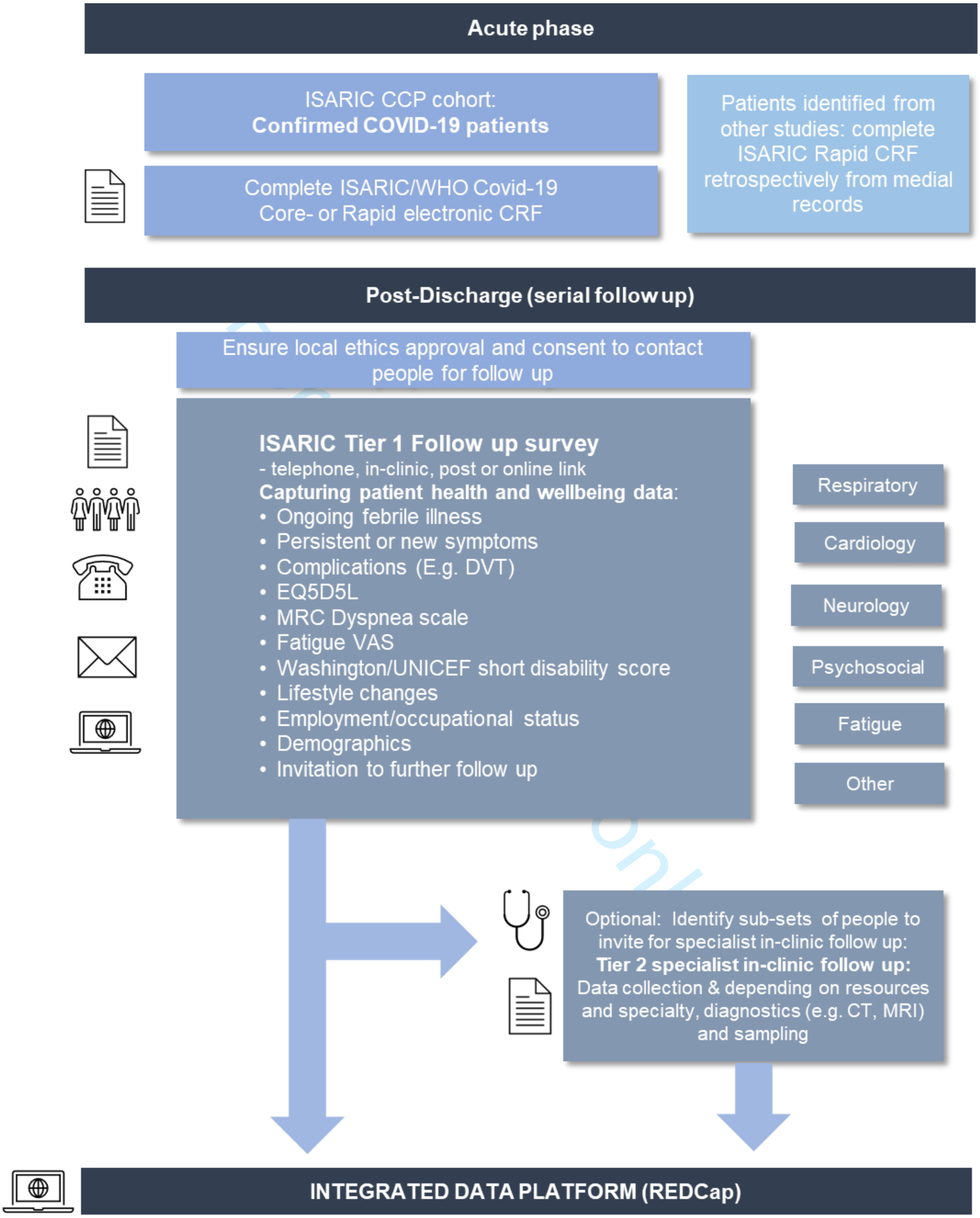
Acute phase
ISARIC CCP cohort:
Confirmed COVID-19 patients
Complete ISARIC/WHO Covid-19
Core- or Rapid electronic CRF
Patients identified from other studies: complete ISARIC Rapid CRF retrospectively from medial records
Post-Discharge (serial follow up)
Ensure local ethics approval and consent to contact people for follow up
ISARIC Tier 1 Follow up survey
- telephone, in-clinic, post or online link
Capturing patient health and wellbeing data:
• Ongoing febrile illness
• Persistent or new symptoms
• Complications (E.g. DVT)
• EQ5D5L
• MRC Dyspnea scale
• Fatigue VAS
• Washington/UNICEF short disability score
• Lifestyle changes
• Employment/occupational status
• Demographics
• Invitation to further follow up
Respiratory
Cardiology
Neurology
Psychosocial
Fatigue
Other
Optional: Identify sub-sets of people to invite for specialist in-clinic follow up:
Tier 2 specialist in-clinic follow up:
Data collection & depending on resources and specialty, diagnostics (e.g. CT, MRI) and sampling
INTEGRATED DATA PLATFORM (REDCap)

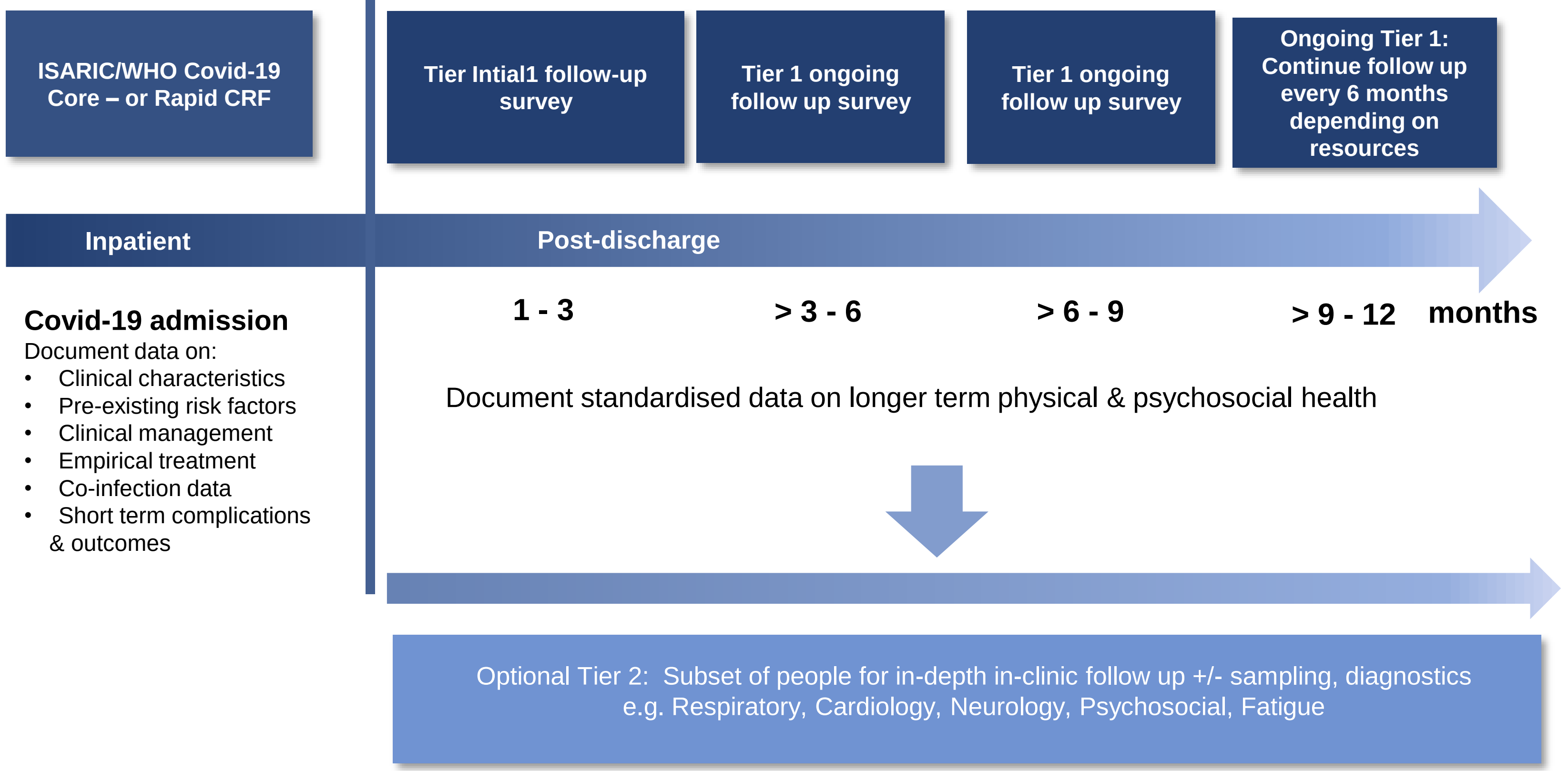
ISARIC/WHO Covid-19 Core – or Rapid CRF
Tier Intial1 follow-up survey
Tier 1 ongoing follow up survey
Tier 1 ongoing follow up survey
Ongoing Tier 1: Continue follow up every 6 months depending on resources
Inpatient
Post-discharge
1 - 3
> 3 - 6
> 6 - 9
> 9 - 12
months
Covid-19 admission
Document data on:
• Clinical characteristics
• Pre-existing risk factors
• Clinical management
• Empirical treatment
• Co-infection data
• Short term complications & outcomes
Document standardised data on longer term physical & psychosocial health
Optional Tier 2: Subset of people for in-depth in-clinic follow up +/- sampling, diagnostics
e.g. Respiratory, Cardiology, Neurology, Psychosocial, Fatigue

BMJ Open

What is the recovery rate and risk of long-term consequences following a diagnosis of COVID-19? – a harmonised, global longitudinal observational study protocol

Journal:	*BMJ Open*
Manuscript ID	bmjopen-2020-043887.R2
Article Type:	Protocol
Date Submitted by the Author:	29-Jan-2021

Complete List of Authors:	Sigfrid, Louise; University of Oxford, ISARIC Global Support Centre, Centre for Tropical Medicine and Global Heatlh<br>Cevik, Muge; University of St Andrews, Infection and Global Health Division, School of Medicine<br>Jesudason, Edwin; NHS Lothian, Rehabilitation Medicine<br>Lim, Wei Shen; Nottingham University Hospitals NHS Trust, Respiratory Medicine<br>Rello, Jordi; Hospital Universitari Vall d'Hebron, Centro de Investigación Biomédica en Red – Enfermedades Respiratorias (CIBERES); Université Nîmes-Montpellier, Research Department, CHU Nîmes<br>Amuasi, John; Kwame Nkrumah University of Science and Technology, School of Public Health<br>Bozza, Fernando; Fundação Oswaldo Cruz<br>Palmieri, Carlo; University of Liverpool, Department of Molecular and Clinical Cancer Medicine, Institute of Systems, Molecular and Integrative Biology; Clatterbridge Cancer Centre NHS Foundation Trust<br>Munblit, Daniel; I M Sechenov First Moscow State Medical University, Paediatrics; Imperial College London Faculty of Medicine, IInflammation, Repair and Development Section, National Heart and Lung Institute<br>Holter, Jan; Oslo University Hospital, Department of Microbiology; University of Oslo, Institute of Clinical Medicine<br>Kildal, Anders Benjamin; University Hospital of North Norway, Department of Anesthesiology and Intensive Care<br>Reyes, Luis; Universidad de La Sabana<br>Russell, Clark; The University of Edinburgh Centre for Inflammation Research<br>Ho, Antonia; University of Glasgow<br>Turtle, Lance; University of Liverpool, NIHR Health Protection Research Unit in emerging and zoonotic infections, Institute of Infection, Veterinary and Ecological Sciences; Liverpool University Hospitals NHS Foundation Trust, Tropical and Infectious Disease Unit<br>Drake, Thomas ; The University of Edinburgh, Centre for Medical Informatics<br>Beltrame, Anna; IRCCS Sacro Cuore Don Calabria Hospital, Department of Infectious Diseases, Tropical and Microbiology<br>Hann, Katrina; Sustainable Health Systems<br>Bangura, Ibrahim; Dorothy Springer Trust<br>Fowler, Robert; Sunnybrook Research Institute, Sunnybrook Health

	Sciences Institute<br>Lakoh, Sulaiman; Sustainable Health Systems<br>Berry, Colin; University of Glasgow, Institute of Cardiovascular and Medical Sciences, BHF Glasgow Cardiovascular Research Centre<br>Lowe, David; Queen Elizabeth University Hospital, Emergency Department<br>McPeake, Joanne; NHS Greater Glasgow and Clyde; University of Glasgow, Institute of Health and Wellbeing<br>Hashmi, Madiha; Ziauddin University, Critical Care Medicine<br>Dyrhol-Riise, Anne Margarita; Oslo University Hospital, Dep. of Infectious Diseases, Institute of Clinical Medicine<br>Donohue, Chloe; University of Liverpool, National Institute of Health Research (NIHR) Health Protection research Unit in Emerging and Zoonotic Infections; University of Liverpool, Institute of Infection and Global Health, Faculty of Health and Life Sciences<br>Plotkin, Daniel; University of Oxford, ISARIC Global Support Centre, Centre for Tropical Medicine and Global Heatlh<br>Hardwick, Hayley; University of Liverpool, National Institute of Health Research (NIHR) Health Protection research Unit in Emerging and Zoonotic Infections; University of Liverpool, Institute of Infection and Global Health, Faculty of Health and Life Sciences<br>Elkheir, Natalie; London School of Hygiene & Tropical Medicine<br>Lone, Nazir; The University of Edinburgh, Usher Institute<br>Docherty, Annemarie; University of Edinburgh, Centre for Medical Informatics, The Usher Institute<br>Harrison, Ewen ; The University of Edinburgh, Director Centre for Medical Informatics, Usher Institute<br>Baille, J; The University of Edinburgh The Roslin Institute, Division of Genetics and Genomics<br>Carson, Gail; University of Oxford, ISARIC Global Support Centre, Centre for Tropical Medicine and Global Health<br>Semple, Malcolm G; University of Liverpool, Health Protection Research Unit In Emerging and Zoonotic Infections, Institute of Infection, Veterinary and Ecological Sciences; Alder Hey Children's NHS Foundation Trust, University of Liverpool<br>Scott, Janet; University of Glasgow Centre for Virus Research, MRC

**Primary Subject Heading**:	Infectious diseases
Secondary Subject Heading:	Global health
Keywords:	COVID-19, INFECTIOUS DISEASES, Public health < INFECTIOUS DISEASES

SCHOLARONE™
Manuscripts

I, the Submitting Author has the right to grant and does grant on behalf of all authors of the Work (as defined in the below author licence), an exclusive licence and/or a non-exclusive licence for contributions from authors who are: i) UK Crown employees; ii) where BMJ has agreed a CC-BY licence shall apply, and/or iii) in accordance with the terms applicable for US Federal Government officers or employees acting as part of their official duties; on a worldwide, perpetual, irrevocable, royalty-free basis to BMJ Publishing Group Ltd ("BMJ") its licensees and where the relevant Journal is co-owned by BMJ to the co-owners of the Journal, to publish the Work in this journal and any other BMJ products and to exploit all rights, as set out in our licence.

The Submitting Author accepts and understands that any supply made under these terms is made by BMJ to the Submitting Author unless you are acting as an employee on behalf of your employer or a postgraduate student of an affiliated institution which is paying any applicable article publishing charge ("APC") for Open Access articles. Where the Submitting Author wishes to make the Work available on an Open Access basis (and intends to pay the relevant APC), the terms of reuse of such Open Access shall be governed by a Creative Commons licence – details of these licences and which Creative Commons licence will apply to this Work are set out in our licence referred to above.

Other than as permitted in any relevant BMJ Author's Self Archiving Policies, I confirm this Work has not been accepted for publication elsewhere, is not being considered for publication elsewhere and does not duplicate material already published. I confirm all authors consent to publication of this Work and authorise the granting of this licence.

For peer review only

What is the recovery rate and risk of long-term consequences following a diagnosis of COVID-19? – a harmonised, global longitudinal observational study protocol

Authors: L. Sigfrid*, M. Cevik*, E. Jesudason, W.S Lim, J. Rello, J. Amuasi, F. Bozza, C.Palmieri, D. Munblit, J.C.Holter, A.B. Kildal, L.F. Reyes, C.D. Russell, A.Ho, L.Turtle, T.M. Drake, A. Beltrame, K. Hann, I.R. Bangura, R. Fowler, S. Lakoh, C. Berry, D.J. Lowe, J. McPeake, M. Hashmi, A.M. Dyrhol-Riise, C.Donohue, D.R. Plotkin, H. Hardwick, N. Elkheir, N. Lone, A. Docherty, E.M. Harrison, J.K. Baille, G. Carson, M.G. Semple, J.T. Scott

*joint first authors

Affiliations:

Louise Sigfrid, PhD Clinical Research Fellow, Public Health Specialist, ISARIC GSC, Centre for Tropical Medicine and Global Health, University of Oxford, Oxford, UK https://orcid.org/0000-0003-2764-1177

Muge Cevik, Infection and Global Health Division, School of Medicine, University of St Andrews, St Andrews, UK https://orcid.org/0000-0003-1133-3874

Edwin Jesudason, Consultant in Rehabilitation Medicine, NHS Lothian, Edinburgh, United Kingdom

Wei Shen Lim, Department of Respiratory Medicine, Nottingham University Hospitals NHS Trust, Nottingham, UK https://orcid.org/0000-0002-7694-3051

Jordi Rello, Prof. 1. Centro de Investigación Biomédica en Red – Enfermedades Respiratorias (CIBERES), Vall d'Hebron Institute of Research (VHIR), Barcelona, Spain. 2. Research Department, CHU Nîmes, Université Nîmes-Montpellier, Nîmes, France. https://orcid.org/0000-0003-0766-6210

John Amuasi (MBChB. MPH. MS. PhD.), School of Public Health, Kwame Nkrumah University of Science and Technology, Ghana https://orcid.org/0000-0002-8640-2662

Fernando Bozza, MD, Fundação Oswaldo Cruz: Rio de Janeiro, RJ, Brasil https://orcid.org/0000-0003-4878-0256

Carlo Palmieri, MD 1. Department of Molecular and Clinical Cancer Medicine, Institute of Systems, Molecular and Integrative Biology, University of Liverpool, UK; 2. The Clatterbridge Cancer Centre NHS Foundation Trust, Liverpool, UK http://orcid.org/0000-0001-9496-2718

Daniel Munblit, Associate Prof. 1. Department of Paediatrics and Paediatric Infectious Diseases, Institute of Child's Health, Sechenov First Moscow State Medical University (Sechenov University), Moscow,

For peer review only

For peer review only - http://bmjopen.bmj.com/site/about/guidelines.xhtml

Russia. 2. Inflammation, Repair and Development Section, National Heart and Lung Institute, Faculty of Medicine, Imperial College London, London, United Kingdom https://orcid.org/0000-0001-9652-6856

Jan Cato Holter, MD, PhD. Specialist in infectious diseases and medical microbiology, 1. Department of Microbiology, Oslo University Hospital, Oslo. 2. Institute of Clinical Medicine, University of Oslo, Oslo. https://orcid.org/0000-0003-1618-5022

Anders Benjamin Kildal, MD, PhD, Specialist in Anesthesiology, Department of Anesthesiology and Intensive Care, University Hospital of North Norway, Tromsø, Norway https://orcid.org/0000-0002-1319-6511

Luis Felipe Reyes, MD, PhD, Universidad de La Sabana, Chía, Colombia, https://orcid.org/0000-0003-1172-6539

Clark D. Russell, MD, Clinical lecturer in Infectious Diseases, Centre for Inflammation Research, University of Edinburgh, UK https://orcid.org/0000-0002-9873-8243

Antonia Ho, MD, PhD, Clinical Senior Lecturer/Consultant in Infectious Diseases, University of Glasgow, Glasgow, UK https://orcid.org/0000-0003-1465-3785

Lance Turtle, MD, PhD, 1. Senior clinical lecturer in infectious diseases, NIHR Health Protection Research Unit in emerging and zoonotic infections, Institute of Infection, Veterinary and Ecological Sciences, University of Liverpool. 2. Consultant in infectious diseases, Tropical and Infectious Disease Unit, Liverpool University Hospitals NHS Foundation Trust. https://orcid.org/0000-0002-0778-1693

Thomas M Drake, MD, PhD, Clinical Research Fellow, Centre for Medical Informatics, University of Edinburgh, Edinburgh, UK, https://orcid.org/0000-0002-9334-6494

Anna Beltrame, MD, Clinician, Department of Infectious Diseases, Tropical and Microbiology, IRCCS Sacro Cuore Don Calabria Hospital: Negrar di Valpolicella, Italy https://orcid.org/0000-0002-7499-5843

Katrina Hann, MD, Sustainable Health Systems, Freetown, Sierra Leone https://orcid.org/0000-0002-4913-6270

Ibrahim Richard Bangura, Data Manager, Dorothy Springer Trust, Sierra Leone

Rob Fowler, MD, PhD. Department of Medicine and Department of Critical Care Medicine, Sunnybrook Hospital, University of Toronto. Toronto, Canada

Sulaiman Lakoh, MD, Sustainable Health Systems, Freetown, Sierra Leone https://orcid.org/0000-0002-7639-0004

For peer review only

Colin Berry, Prof. Institute of Cardiovascular and Medical Sciences, BHF Glasgow Cardiovascular Research Centre, University of Glasgow, Glasgow, UK, https://orcid.org/0000-0002-4547-8636

David J Lowe, MD,Emergency Department, Queen Elizabeth University Hospital, Glasgow, Scotland, UK https://orcid.org/0000-0003-4866-2049

Joanne McPeake, 1. NHS Greater Glasgow and Clyde 2. University of Glasgow Institute of Health and Wellbeing 3. THIS Institute, University of Cambridge ORCID: https://orcid.org/0000-0001-8206-6801

Madiha Hashmi, 1. Critical Care Medicine, Ziauddin University, Karachi, Pakistan 2. Pakistan Registry of Intensive CarE (PRICE), Pakistan. 3. South East Asian Research in Critical care Health (SEARCH).

Dyrhol-Riise Anne Margarita, MD, Prof. Dep. of Infectious Diseases, Oslo University hospital, Institute of Clinical Medicine, University of Oslo, Norway. https://orcid.org/0000-0003-4009-4032

Chloe Donohue, 1. National Institute of Health Research (NIHR) Health Protection research Unit in Emerging and Zoonotic Infections, Liverpool, UK. 2. Institute of Infection and Global Health, Faculty of Health and Life Sciences, University of Liverpool, Liverpool, UK

Daniel R Plotkin, ISARIC Global Support Centre, Centre for Tropical Medicine and Global Health, Nuffield Department of Medicine, University of Oxford, Oxford, UK https://orcid.org/0000-0001-9454-1382

Hayley Hardwick, 1. National Institute of Health Research (NIHR) Health Protection research Unit in Emerging and Zoonotic Infections, Liverpool, UK. 2. Institute of Infection and Global Health, Faculty of Health and Life Sciences, University of Liverpool, Liverpool, UK https://orcid.org/0000-0003-3421-6435

Natalie Elkheir, MD, Clinical research fellow, London School of Hygiene and Tropical Medicine, London, UK https://orcid.org/0000-0002-1786-8530

Nazir Lone, MD, Usher Institute, Edinburgh, UK https://orcid.org/0000-0003-2707-2779

Annemarie Docherty, MD, 1. Centre for Medical Informatics, Usher Institute, University of Edinburgh, Edinburgh, UK. 2. [2]Intensive Care Unit, Royal Infirmary Edinburgh, Edinburgh, UK

Ewen M Harrison, Prof. Director Centre for Medical Informatics, Usher Institute, University of Edinburgh, Edinburgh, UK https://orcid.org/0000-0002-5018-3066

J Kenneth Baillie, MD, Roslin Institute, University of Edinburgh, Edinburgh, UK ORCID:0000-0001-5258-793X

Gail Carson, ISARIC, Centre for Tropical Medicine and Global Health, Nuffield Department of Medicine, University of Oxford, Oxford, UK https://orcid.org/0000-0001-8439-9933

For peer review only

For peer review only - http://bmjopen.bmj.com/site/about/guidelines.xhtml

Malcolm G. Semple. Prof. Health Protection Research Unit In Emerging and Zoonotic Infections, Institute of Infection, Veterinary and Ecological Sciences, University of Liverpool, UK 2. Respiratory Medicine, Alder Hey Children's NHS Foundation Trust, Eaton Road, Liverpool, UK

https://orcid.org/0000-0001-9700-0418

Janet T Scott, MD, PhD, MRC-University of Glasgow Centre for Virus Research, Glasgow, UK

https://orcid.org/0000-0001-8030-5223

Corresponding author:

Louise Sigfrid, MD, PhD, ISARIC Global Support Centre, NDM, Centre for Tropical Medicine and Global Health, University of Oxford, Oxford, UK

E-mail: louise.sigfrid@gmail.com

Abstract:

Introduction

Very little is known about possible clinical sequelae that may persist after resolution of the acute Coronavirus Disease 2019 (COVID-19). A recent longitudinal cohort from Italy including 143 patients recovered after hospitalisation with COVID-19 reported that 87% had at least one ongoing symptom at 60-day follow-up. Early indications suggest that patients with COVID-19 may need even more psychological support than typical ICU patients. The assessment of risk factors for longer term consequences requires a longitudinal study linked to data on pre-existing conditions and care received during the acute phase of illness. The primary aim of this study is to characterise physical and psychosocial sequelae in patients post-COVID-19 hospital discharge.

Methods and analysis

This is an international open-access prospective, observational multi-site study. This protocol is linked with the International severe acute respiratory and emerging infection consortium (ISARIC) and the WHO's clinical characterisation protocol, which includes patients with suspected or confirmed COVID-19 during hospitalisation. This protocol will follow up a subset of patients with confirmed COVID-19 using standardised surveys to measure longer term physical and psychosocial sequelae. The data will be linked with the acute phase data. Statistical analyses will be undertaken to characterise groups most

For peer review only

likely to be affected by sequelae of COVID-19. The open-access follow up survey can be used as a data collection tool by other follow up studies, to facilitate data harmonisation, and to identify sub-sets of patients for further in-depth follow up. The outcomes of this study will inform strategies to prevent long term consequences; inform clinical management, interventional studies, rehabilitation, and public health management to reduce overall morbidity and improve long term outcomes of COVID-19.

Ethics and dissemination

The protocol and survey are open-access to enable low resourced sites to join the study, to facilitate global standardized, longitudinal data collection. Ethical approval has been given by sites in Colombia, Ghana, Italy, Norway, Russia, the UK and S. Africa. New sites are welcome to join this collaborative study at any time. Sites interested in adopting the protocol as it is or in an adapted version are responsible for ensuring that local sponsorship and ethical approvals in place as appropriate. The tools are available on the ISARIC website (www.isaric.org).

Protocol registration number: osf.io/c5rw3/

Protocol version: 3 August 2020

EuroQol ID: 37035

Key words: COVID-19, post-acute COVID-19 sequelae, long-term consequences, SARS-CoV-2, observational study protocol, open-access, pandemic research

For peer review only - http://bmjopen.bmj.com/site/about/guidelines.xhtml

Article summary

Strengths and limitations of this study

- This is an open-access protocol and data collection forms to facilitate standardised, multisite data collection to forward knowledge into long term consequences of COVID-19.
- This study aims to inform strategies to prevent longer term sequalae; inform clinical management, rehabilitation, and public health management strategies to reduce morbidity and improve outcomes.
- The protocol will be used to follow up a sub-set of patients, already included in the existing ISARIC cohort of more than 95,966 individuals hospitalized with confirmed COVID-19 across 42 countries (as of 20 Nov. 2020).
- The follow up data will be linked with acute-phase data already documented using the ISARIC/WHO standardized Core- or RAPID case report forms (CRFs).
- The data collection tool is developed to facilitate wide dissemination and uptake with limited resources, to mitigate resources limitations during the pandemic.

Introduction

Coronavirus Disease 2019 (COVID-19), caused by Severe Acute Respiratory Syndrome Coronavirus-2 (SARS-CoV-2) infection, can lead to a diverse range of clinical manifestations, ranging from an asymptomatic infection to an acute respiratory distress syndrome, and multiorgan failure with high risk of mortality (1). It is established that SARS-CoV-2 not only infects the respiratory tract but that ensuing viral replication and immune response may also affect other organs, which can lead to a risk of heart, renal and liver injury, in addition to an acute systemic inflammatory response and accompanying circulatory shock (2-4). While most people have uncomplicated recoveries, some have prolonged illness even after recovery from the acute illness (5-7). Identifying longer-term potential consequences and relationship with the acute illness is important for the management of patients, in particular, understanding how these interact and affect those already living with other conditions such as cardiovascular disease and cancer will be paramount.

For peer review only - http://bmjopen.bmj.com/site/about/guidelines.xhtml

However, very little is known about possible clinical sequelae that may persist after the resolution of acute infection. A recent longitudinal cohort of 143 patients followed after hospitalisation from COVID-19 in Italy, reported that 87% had at least one ongoing symptom, most (55%) with 3 or more symptoms at 60 day follow up, fatigue (53%), dyspnoea (43%), joint pain (27%) and chest pain (22%) being the most common. COVID-19 was associated with worsened quality of life among 44% of patients. (6) Prolonged course of illness has also been reported among people with mild COVID-19 who did not require hospitalisation (5, 7, 8).

Increasing evidence also suggests that infection with SARS-CoV-2 can cause neurological consequences, (2) including altered mental status, comprising encephalopathy or encephalitis and primary psychiatric diagnoses (9). While these symptoms arise acutely during the course of infection, less is known about the possible long-term consequences. Severely affected COVID-19 cases experience high levels of proinflammatory cytokines and acute respiratory dysfunction which often require assisted ventilation. These are known factors suggested to cause cognitive decline (2, 10).

Post-traumatic stress disorder (PTSD) and post-intensive care syndrome after intensive care unit (ICU) stay has been well documented previously (11-13). A systematic review of consequences after hospitalisation or ICU stay for severe acute respiratory infection (SARS-CoV) and Middle East respiratory syndrome coronavirus (MERS-CoV) found sequelae up to 6 months after discharge. Common consequences besides impaired diffusing capacity for carbon monoxide and reduced exercise capacity were PTSD (39%), depression (33%) and anxiety (30%) (14). Additionally, serial CT scans post discharge after SARS-CoV showed a gradual healing of pulmonary injury, with pulmonary consequences lasting more than 6 months post discharge. (15)

Early indications suggest patients with COVID-19 will need even more psychological support than typical post-ICU patients because of higher levels of “survivors’ guilt” and PTSD (16). In addition, the characteristics of the initial cellular immune and antibody response to SARS-CoV-2 have not been fully defined and it is not known if the immune responses generated by infection provides long-term protective immunity. Identifying multidisciplinary sequalae and complications through high quality, global studies

For peer review only - http://bmjopen.bmj.com/site/about/guidelines.xhtml

throughout the course of COVID-19 is important for the acute and longer-term management of patients (4, 17). The emerging data and anecdotal evidence of long-term recovery and persistent debilitating symptoms highlight the need for robust, standardised studies to assess the risk of and risk factors for COVID -19 sequalae.

The purpose of this study is to establish a longitudinal cohort of patients with COVID -19 post-discharge to characterise the risk of long-term consequences over time in different populations globally. (18) The primary outcome is to characterise physical and psychosocial consequences in patients post-COVID- 19 infection. Secondary outcomes include estimating the risk of and risk factors for post-COVID- 19 medical sequalae, psychosocial consequences and long-term outcomes. The results will inform strategies to prevent long term consequences; inform clinical management, interventional research, direct rehabilitation, and inform public health management to reduce overall morbidity and improve outcomes of COVID-19. This study is open access for any sites globally to join the collaboration to characterise COVID-19 in different populations. Providing research tools that can be implemented for free in low resourced settings can increase equity in implementation of and inclusion in clinical research studies.

Methods and analysis

This protocol and data collection surveys have been developed by the ISARIC COVID-19 global follow up working group and informed by a wide range of global stakeholders with expertise in clinical research, outbreak research, infectious disease, epidemiology, respiratory, critical care, rehabilitation, neurology, psychology, rheumatology, cardiology, oncology and public health medicine.(18)

Study design

This is an international prospective, observational multi-site cohort study to assess risk of and risk factors for longer term physical and psychosocial consequences of COVID-19. The study conforms to research ethics standards and has been approved by research ethics boards in Colombia, Ghana, Italy, Norway and the UK and submitted for ethics approval in additional sites and countries. All sites adopting the protocol are responsible for ensuring that they have local ethical approval in place.

For peer review only - http://bmjopen.bmj.com/site/about/guidelines.xhtml

Population and setting

This protocol builds on the ISARIC/WHO COVID-19 clinical characterisation protocol (CCP) and associated data collection forms already in operation, the Core- and Rapid case report forms (CRFs).(19) These CRFs were developed to standardised clinical data collection on patients admitted with suspected or confirmed COVID-19, with clinical data on more than 95,966 individuals hospitalized with confirmed COVID-19 infection across 42 countries documented in a central database (as of 20 Nov. 2020). (20) These CRFs collect data on demographics, pre-existing comorbidities and risk factors, signs and symptoms experienced during the acute phase, and care and treatments received during hospitalization. (19)

This acute phase data will be linked with the follow up data to inform analysis. A subset of these patients with confirmed COVID-19, will be followed up over time, documenting data on longer term consequences using the Tier 1 follow up data collection surveys.(18) There is no limit on the number of sites or countries taking part.

New clinical sites are invited to take part in this global collaborative effort and use these open-access tools. New sites can complete the Core- or RAPID- CRFs prospectively or retrospectively (Figure 1). Sites adopting the protocol and tools for collaborative or independent studies are responsible for ensuring local regulatory and ethical approvals are in place as appropriate.

Specific inclusion and exclusion criteria for the follow up cohort are as follows:

Inclusion criteria

- People aged 16 years and older
- Laboratory or physician confirmed COVID -19
- At least 1 month post- discharge from hospital or health centre
- Person (or family member/carer for patients who lack capacity) consent to participate

Inclusion of vulnerable participants

The data collection surveys and validated tools are developed for anyone who fit the inclusion criteria, including pregnant women, elderly and those who are immunosuppressed. An aligned study protocol and survey will be developed for following up children.

For peer review only - http://bmjopen.bmj.com/site/about/guidelines.xhtml

Outcomes and Procedure

The follow up protocol and survey are designed to be flexible in a tiered approach to be adapted depending on local resources and research needs. The Tier 1 survey is designed to enable patient self-assessment to facilitate distribution to all patients that fit the inclusion criteria, using a range of methods via post, an online link for self-completion or via telephone or in-clinic completion. A combination of methods can be used to be as inclusive as possible and depending on site resources. The Tier 1 survey designed can be used to identify people with set symptoms for further in-depth in clinic analysis (Tier 2) . The Tier 1 survey is open access for sites to adopt as it is or adapt and combine with sampling and diagnostic methods to support further analysis (Figure 1).

Figure 1. ISARIC's adaptable Covid-19 Follow-Up Protocol framework

Abbreviations: ISARIC: International Severe Acute Respiratory and emerging Infection Consortium, MRC: The Medical Research Council, VAS: Visual analogues scale, WHO: World Health Organisation

Serial follow up

The aim is to follow up patients serially over time at regular intervals, for as long as there is a need and resources. The Tier 1 initial survey will be used at the inital follow up timepoint (at 1 to 3 months post discharge). The Tier 1 ongoing survey will be used at subsequent follow up time points every 3 to 6 months depending on resources (Figure 2). To faciliate combined analysis the time frames in Figure 2 are recommended, however, these can be adapted depending on local resources and needs. For sites that have capacity, the Tier 1 form can be used to identify people for more in-depth in clinic follow up.By being designed to enable patient self-completion, it allows wide distribution at low resource need. It can also be completed via in-clinic or telephone assessments during check-ups or for patients that are still hospitalised. The module will collect data on demographics, hospital stay and re-admissions, all-cause and cause specific mortality (after the initial index event), specific consequences including; deep vein thrombosis (DVT), pulmonary embolism, recent febrile illness, new or persistent symptoms, quality of life (measured by EQ-5D-5L), dyspnoea (assessed using MRC dyspnoea scale), difficulties in functioning (UN/Washington disability score), lifestyle and socioeconomic data.(18) Medical records will be used to

For peer review only - http://bmjopen.bmj.com/site/about/guidelines.xhtml

assess if a person is deceased before each follow up time point, and documented in the database. The ISARIC collaborative follow up study is registered with EuroQol. Sites who wish to adopt the survey for independent studies, outside of the ISARIC collaboration need to register to use the EQ-5D-5L tool with EuroQol.

Sub-studies

The Tier 1 follow up module can be used to identify sub-sets of patients experiencing specific symptomatology or syndromes for further follow up (Tier 2). By using a tiered approach, additional specialist modules can be added for more complex follow up of emerging consequences in a flexible, adaptable way, of a subset of patients, and combined with sampling and diagnostics (Figure 1 and 2).

Figure 2. Schematic overview of the follow up data time-frames

Serial follow up will continue beyond 12 months at 3 to 6 months' interval for up to 3 years depending on resources.

Abbreviations: ISARIC: International Severe Acute Respiratory and emerging Infection Consortium, WHO: World Health Organisation

Biological samples

The Tier 1 surveys can be used on its own for data collection, or in combination with sampling (e.g. respiratory samples, serum, blood, stool, urine), for immunology, pathophysiology, genomics or other studies (16). This protocol builds on the ISARIC/WHO Clinical Characterisation Protocol (CCP) which includes an adaptable research sample schedule for sites with resources for research sampling and analysis (19, 21). The CCP is designed to enable a harmonized, multisite clinical observational research response to any severe or potentially severe acute infection of public health interest, such as COVID-19. It is a standardized protocol for data and biological samples to be collected rapidly in a globally harmonised manner.(21, 22) The CCP was activated in January 2020 to respond to Covid-19 worldwide It is designed in a tiered approach and includes different levels of sampling schedules (acute phase and follow up) that can be adapted depending on resources, to be combined with patient data collection using the acute phase CRFs and the follow up CRF. (18, 19)

For peer review only - http://bmjopen.bmj.com/site/about/guidelines.xhtml

Outcomes

The primary outcome of this study is to characterise physical and psychosocial consequences in patients post-acute COVID- 19. Secondary outcomes include estimating the risk of and risk factors for post-COVID- 19 medical sequalae, psychosocial consequences and post-COVID-19 mortality.

Data collection and entry

A standardised COVID-19 follow up survey aimed for global settings was developed through a series of virtual working group meetings and e-mail iterations. The survey was piloted on patients in four settings in three countries and feedback incorporated into the final form. The form collects data on a wide range of outcomes including hospital stay and all-cause and cause specific mortality (after the initial index event), new or persistent symptoms and complications, e.g. DVT, pulmonary embolism, recent febrile illness, new, persistent symptoms, EQ-5D-5L, MRC dyspnoea scale, UN/Washington disability score, lifestyle and employment data. By standardising data collection, and providing open-access, adaptable tools, focused on key data variables, it can optimize data quality and reduce the burden of research on staff, while collecting the most relevant information to inform clinical care guidelines and public health. This Tier 1 follow up survey is designed for patient self-assessment, via online link or paper form, or to be completed during in-clinic or telephone follow-up appointment in settings globally (Figure 1). (18) The CRF is available open access on the ISARIC website (https://isaric.org/research/covid-19-clinical-research-resources/covid-19-long-term-follow-up-study/) and as an electronic form on the ISARIC hosted REDCap database. (18)

Statistical analysis plan

Using the data, we will test for differences in outcomes across important demographic groups (age categories, sex, ethnicity, socioeconomic deprivation, comorbidities), specific exposures (severe COVID-19, critical care admission, ventilation) and initial clinical sequelae (complications on their index admission for COVID-19). We plan to use this platform to conduct timely analyses which coincide with public health or scientific need. Given these requirements, new questions we have not specified within this protocol may arise. Where this occurs, we will develop analysis plans prior to undertaking analyses, which will be made available on request. The data collected through the follow up module will be linked

For peer review only - http://bmjopen.bmj.com/site/about/guidelines.xhtml

with data on demographics, comorbidities, clinical characteristics, care and treatments collected using the ISARIC/WHO Core- or RAPID COVID-19 CRF.(19)

Fields contained within the data collection forms will be combined and if an area of interest is found, the maximal amount of data will be used to investigate this to maintain sample size and power. The plan below presents our guiding statistical framework. Analyses will be developed concurrently with data collection using statistical coding script. At appropriate time intervals, scripts will be run to produce analyses at these timepoints over the course of the project.

As COVID-19 is a new disease, there are no systematically collected long-term data to base formal sample size calculations upon. Therefore, we intend to recruit as many patients as possible. Through the network established already, this is anticipated to be very large. Therefore, as a minimum calculation, to perform logistic regression, we will use at least 10 events for each variable included in the regression. For a regression containing 10 explanatory variables, 100 events would be included within the model for each variable. Assuming a sequelae rate of at least 20%, we would need at least 500 patients which should be adequately achieved. As the field is rapidly evolving, the analyses are likely to change.

Entered data will be summarised first by using simple summary statistics. Categorical data will be explored using frequencies and percentages, with differences in disease severity and treatment groups tested for using Chi-square tests or fisher's exact test where cell counts are under five. For continuous data, distribution will be established using histograms and density plots. Data that are normally distributed will be summarised using group mean averages and standard deviation as a measure of central tendency. For non-parametric data, the median average will be used and presented alongside 25th and 75th centiles. Differences in normally distributed continuous data will be tested using Welch's two sample t-tests for 2 group data and ANOVA for three or more groups. Mann-Whitney U will be used to compare differences across two groups or Kruskall-Wallis tests for three or more groups, where data follow a non-parametric distribution.

For peer review only - http://bmjopen.bmj.com/site/about/guidelines.xhtml

Outcomes will be expressed in three ways; 1) binary event data (for presence or absence of outcome of interest), 2) change over time (for continuous or ordinal data), or as 3) time to event data (for patients with serial measurements). We will calculate changes over time across symptom and outcome variables and use these changes over time to compare the effect of treatments or exposures on these outcomes. Time to event data will be captured for those who complete serial assessment forms. These data will be presented as Kaplan-Meier plots and differences tested for using log-rank tests. Competing risks (including death) will be accounted for using censoring.

To address the main aim of characterising the middle to long-term impact of COVID-19 on physical and mental health, we will first provide simple summaries of incidence and second, characterise which patients are at risk of developing these. To identify which patients are likely to develop persistent complications, functional impairment or reduced quality of life, we will use multilevel models to adjust for potential confounders. Level I fixed effects will include patient-level explanatory variables (i.e. age, sex) and level II or III random effects will include site and country in the case where research questions require differences in country to be accounted for. Explanatory variables will be entered into models since clinical plausibility and final model selection guided by maximisation of the adjusted R^2 value and minimisation of the Akaike information criterion (AIC) or Bayesian information criterion (BIC). For binary event data, multilevel logistic regression will be used, and estimates presented as odds ratios alongside the corresponding 95% confidence interval. For continuous data, linear or generalised linear regression will be used, and estimates presented as model coefficients, with 95% confidence intervals. Finally, time to event data will be presented as survival probability or hazard ratios, with 95% confidence intervals.

Statistical significance will be taken at the level of $P < 0.05$ *a-priori*. Analyses will be conducted in secure R (R Foundation for Statistical Computing, Vienna, AUT) or STATA (StataCorp LLC, TX, USA) environments.

Data sharing

Sites who wish to utilise ISARIC's COVID-19 data management and hosting support can email ncov@isaric.org to gain access to a secure data capture and management system. All systems are free

For peer review only - http://bmjopen.bmj.com/site/about/guidelines.xhtml

For peer review only

to use and supported by ISARIC data management specialists. Sites who submit data to ISARIC will sign a Terms of Submission, enabling use of the data in collaborative analysis by ISARIC partners. Data entered into the ISARIC database are stored on the servers administered by University of Oxford Medical Sciences Division Information Technology Services (MSD ITS). Data are stored in two active primary data storage locations (Data Centres) which host mirrored copies of data, one located near the MSD ITS offices at the John Radcliffe Hospital and the second at the Old Road Campus Research Building (sites A and B respectively, both in Headington, Oxford, UK).

Patient and public involvement

This protocol and the data collection survey has been informed by people living with long Covid, It has been piloted with a number of patients in different settings, and feedback incorporated in the final version. This included suggestions on the data on symptoms collected and the way questions were asked as well as on the patient information.

Ethics and dissemination

The protocol and survey are open-access to enable low resourced sites to join the study, to facilitate global standardized, longitudinal data collection. The tools (18) are available in a range of languages. Data from combined analysis will be disseminated through the ISARIC website and in open-access publications under group authorship. Ethical approval has been given by the Universidad de La Sabana's IRB, Colombia; Ghana Health Service Ethics Review Committee and at the Comitato Etico di Brescia, Italy, South-Eastern Norway Regional Health Authority, Norway, the Comitato Etico per la sperimentazione Clinica delle Province di Verona e Rovigo, Italy, the Sechenov University Ethics Committee, Moscow, Russian Federation and the University of Witwatersrand Human Research Ethics Committee (Medical), Johannesburg, South Africa. In the UK, for day – 28 follow up as part of the UK CCP approved by the South Central - Oxford C Research Ethics Committee in England and the Scotland A Research Ethics Committee. New sites are welcome to join this collaborative study at any time. Sites interested in adopting the protocol as it is or in an adapted version are responsible for ensuring that local sponsorship and ethical approvals in place as appropriate.

For peer review only - http://bmjopen.bmj.com/site/about/guidelines.xhtml

Data statement

ISARIC stands by the principles of data sharing in public health emergencies. ISARIC supported studies will share quality data in a timely, valid, and governed manner to inform public health policy and benefit patient care. The ISARIC-hosted data platform enables rapid and harmonised operationalisation of data collection to a secure database (23). Ownership and control of the data entered are retained by those who enter the data. Sites can contribute data to combined analysis upon permission. Technical appendix, statistical code and datasets are available from https://isaric.org/document/covid-19-data-management-hosting/ or by contacting: ncov@isaric.org. The data contributors will share the outcome results with key stakeholders to inform public health response, policy development and implementation. To efficiently make findings from this project visible and accessible to a wide range of stakeholders as well as to networks of individuals and institutions, we will utilise social media, including the ISARIC and partner institution's websites. The consortium will manage a page dedicated to this project that will incorporate all the tools available. The webpage will provide not only updates on the progress of the project and component activities and events, but also information on research.

This follow up study is developed as an open-access tool to be adopted as appropriate by any site interested in following up patients with COVID-19 over time, to facilitate standardiszed data collection globally to enable combined analysis.New sites globally are invited to join the study at any time. The outcomes of this study will inform strategies to prevent risk of consequences; clinical management, rehabilitation and public health management needs to reduce morbidity and improve outcomes. We invite hospitals and healthcare centres globally to collaborate and take part in the study.

Acknowledgments

We would like to thank the ISARIC global clinical characterisation group, ISARIC4C and all the clinicians, nurses and researchers contributing COVID-19 clinical patient data, which will be linked with the follow up data, and all the patients that have consented to be followed up. We would like to acknowledge WHO, whose working groups contributed to development of the CCP and associated tools. Moreover, Sarah Moore, Romans Matulevics, James Lee, Laura Merson, Peter Bannister, Katherine Maskell, Eli

For peer review only - http://bmjopen.bmj.com/site/about/guidelines.xhtml

Harriss, Liliana Resende, Anneli Sandström and Tova Strong for administrative, graphic design and dissemination support.

Author contributions: JTS and LS lead on the development of the follow up protocol and tools (survey , consent form and patient information sheet) in collaboration with members of the ISARIC COVID-19 follow up working group (JR, JA, FB, CP, DM, JCH, ABK, CDR, TMD, A-MDR, KH, IRB, AB, MH, RF, SL, LFR , AH, CD, HH, NE, LT, CR, MC, WSL) and external specialists (EJ, DJL, CB, JM, NL, MGS, JKB, EMH, AD, GC) through a series of meetings and e-mail iterations. AH, LT, ABK, JR, CP piloted the case report form in the clinic, before it was finalised by the members of the working group. TMD, LFR, LS, JTS, AD, EMH developed the statistical analysis plan. DRP, HH and CD managed the database set up on REDCap and individual site set up. LS, MC, TMD and JTS lead on drafting of the protocol and manuscript, with contributions from (JR, JA, FB, CP, DM, JCH, ABK, CDR, TMD, A-MDR, KH, IRB, AB, MH, RF, SL, LFR , AH, CD, HH, NE, LT, CR, MC, WSL, EJ, DJL, CB, JM, NL, MGS, JKB, EMH, AD, GC). All authors reviewed and approved the final manuscript.

Word count: 4005

Competing interest statement: No competing interests declared.

Ethical approval: Ethical approval has been given by the Universidad de La Sabana's IRB (US-MED-504), Colombia; Ghana Health Service Ethics Review Committee and at the Comitato Etico di Brescia, Italy, South-Eastern Norway Regional Health Authority, Norway (Ref 106624). In the UK, for day – 28 follow up as part of the UK CCP approved by the South Central - Oxford C Research Ethics Committee in England (Ref 13/SC/0149) and the Scotland A Research Ethics Committee (Ref 20/SS/0028). The protocol is under review at the Comitato Etico per la sperimentazione Clinica delle Province di Verona e Rovigo, Italy, the Sechenov University Ethics Committee, Moscow, Russian Federation and the University of Witwatersrand Human Research Ethics Committee (Medical), Johannesburg, South Africa and is being submitted to the National Ethics Committee (CONEP), Rio de Janeiro, Brazil, and in Freetown, Sierra Leone. Sites interested in adopting the protocol as it is or in an adapted version are responsible for ensuring that local sponsorship and ethical approvals in place as appropriate.

For peer review only

For peer review only - http://bmjopen.bmj.com/site/about/guidelines.xhtml

Funding statement: This work was supported by the Department for International Development and Wellcome [215091/Z/18/Z] and the Bill & Melinda Gates Foundation [OPP1209135]. CP would like to acknowledge the support of the Liverpool Experimental Cancer Medicine Centre (Grant Reference: C18616/A25153) and The Clatterbridge Cancer Centre Charity. CB acknowledges the support from British Heart Foundation RE/18/6134217.

For peer review only

References

1. Cevik M, Bamford CGG, Ho A. COVID-19 pandemic-a focused review for clinicians. Clin Microbiol Infect. 2020;26(7):842-7.
2. Heneka MT, Golenbock D, Latz E, Morgan D, Brown R. Immediate and long-term consequences of COVID-19 infections for the development of neurological disease. Alzheimer's research & therapy. 2020;12(1):69.
3. Connors J, Levy J. COVID-19 and its implications for thrombosis and anticoagulation. Blood. 2020;135(23):2033-40.
4. Zaim S, Chong JH, Sankaranarayanan V, Harky A. COVID-19 and Multiorgan Response. Current problems in cardiology. 2020;45(8):100618.
5. Rayner C, Lokugamage A, Molokhia M. Covid-19: Prolonged and relapsing course of illness has implications for returning workers BMJ. 2020.
6. Carfì A, Bernabei R, Landi F, Group ftGAC-P-ACS. Persistent Symptoms in Patients After Acute COVID-19. JAMA. 2020.
7. Tenforde MW, Kim SS, Lindsell CJ, Rose EB, Shapiro NI, Files DC, et al. Symptom Duration and Risk Factors for Delayed Return to Usual Health Among Outpatients with COVID-19 in a Multistate Health Care Systems Network — United States, March–June 2020 2020 [
8. Assaf G, Davis H, McCorkell L, Wei H, O'Neill B, Akrami A, et al. What Does COVID-19 Recovery Actually Look Like? An Analysis of the Prolonged COVID-19 Symptoms Survey by Patient-Led Research Team. London, UK: The COVID-19 Body Politic Slack Group; 2020. Contract No.: 2 May 2020.
9. Varatharaj A, Thomas N, Ellul MA, Davies NWS, Pollak TA, Tenorio EL, et al. Neurological and neuropsychiatric complications of COVID-19 in 153 patients: a UK-wide surveillance study. The lancet Psychiatry. 2020.
10. Sasannejad C, Ely EW, Lahiri S. Long-term cognitive impairment after acute respiratory distress syndrome: a review of clinical impact and pathophysiological mechanisms. Crit Care. 2019;23(1):352-.
11. Parker AM, Sricharoenchai T, Raparla S, Schneck KW, Bienvenu OJ, Needham DM. Posttraumatic stress disorder in critical illness survivors: a metaanalysis. Critical care medicine. 2015;43(5):1121-9.
12. Davydow DS, Gifford JM, Desai SV, Needham DM, Bienvenu OJ. Posttraumatic stress disorder in general intensive care unit survivors: a systematic review. General hospital psychiatry. 2008;30(5):421-34.
13. Bangash MN, Owen A, Alderman JE, Chotalia M, Patel JM, Parekh D. COVID-19 recovery: potential treatments for post-intensive care syndrome. The Lancet Respiratory Medicine. 2020;8(11):1071-3.
14. Ahmed H, Patel K, Greenwood DC, Halpin S, Lewthwaite P, Salawu A, et al. Long-term clinical outcomes in survivors of severe acute respiratory syndrome and Middle East respiratory syndrome coronavirus outbreaks after hospitalisation or ICU admission: A systematic review and meta-analysis. Journal of rehabilitation medicine. 2020;52(5):jrm00063.
15. Salehi S, Reddy S, Gholamrezanezhad A. Long-term Pulmonary Consequences of Coronavirus Disease 2019 (COVID-19): What We Know and What to Expect. Journal of thoracic imaging. 2020;35(4):W87-w9.
16. Thornton J. Covid-19: the challenge of patient rehabilitation after intensive care. BMJ. 2020;369:m1787.
17. Holmes EA, O'Connor RC, Perry VH, Tracey I, Wessely S, Arseneault L, et al. Multidisciplinary research priorities for the COVID-19 pandemic: a call for action for mental health science. The Lancet Psychiatry. 2020;7(6):547-60.
18. ISARIC. ISARIC Covid-19 long term follow up study Oxford, UK2020 [cited 2020 13 Nov. 2020]. Available from: https://isaric.org/research/covid-19-clinical-research-resources/covid-19-long-term-follow-up-study/.
19. ISARIC. ISARIC/WHO Core- and Rapid- Covid-19 Case Report Forms Oxford, UK2020 [cited 2020 13 Nov.]. Available from: https://isaric.org/research/covid-19-clinical-research-resources/covid-19-crf/.
20. Pritchard M, Dankwa EA, Hall M, Baillie JK, Carson G, Docherty A, et al. ISARIC Clinical Data Report 4 October 2020. medRxiv. 2020:2020.07.17.20155218.

For peer review only

For peer review only - http://bmjopen.bmj.com/site/about/guidelines.xhtml

21. ISARIC, WHO. Clinical Characterisation Protocol (CCP): ISARIC; 2012 [cited 2020 13 Nov.]. version 3.2:[Available from: https://isaric.org/research/covid-19-clinical-research-resources/clinical-characterisation-protocol-ccp/.

22. Akhvlediani T, Ali SM, Angus DC, Arabi YM, Ashraf S, Baillie JK, et al. Global outbreak research: harmony not hegemony. The Lancet Infectious Diseases. 2020;20(7):770-2.

23. Harris PA, Taylor R, Thielke R, Payne J, Gonzalez N, Conde JG. Research electronic data capture (REDCap)--a metadata-driven methodology and workflow process for providing translational research informatics support. Journal of biomedical informatics. 2009;42(2):377-81.

For peer review only

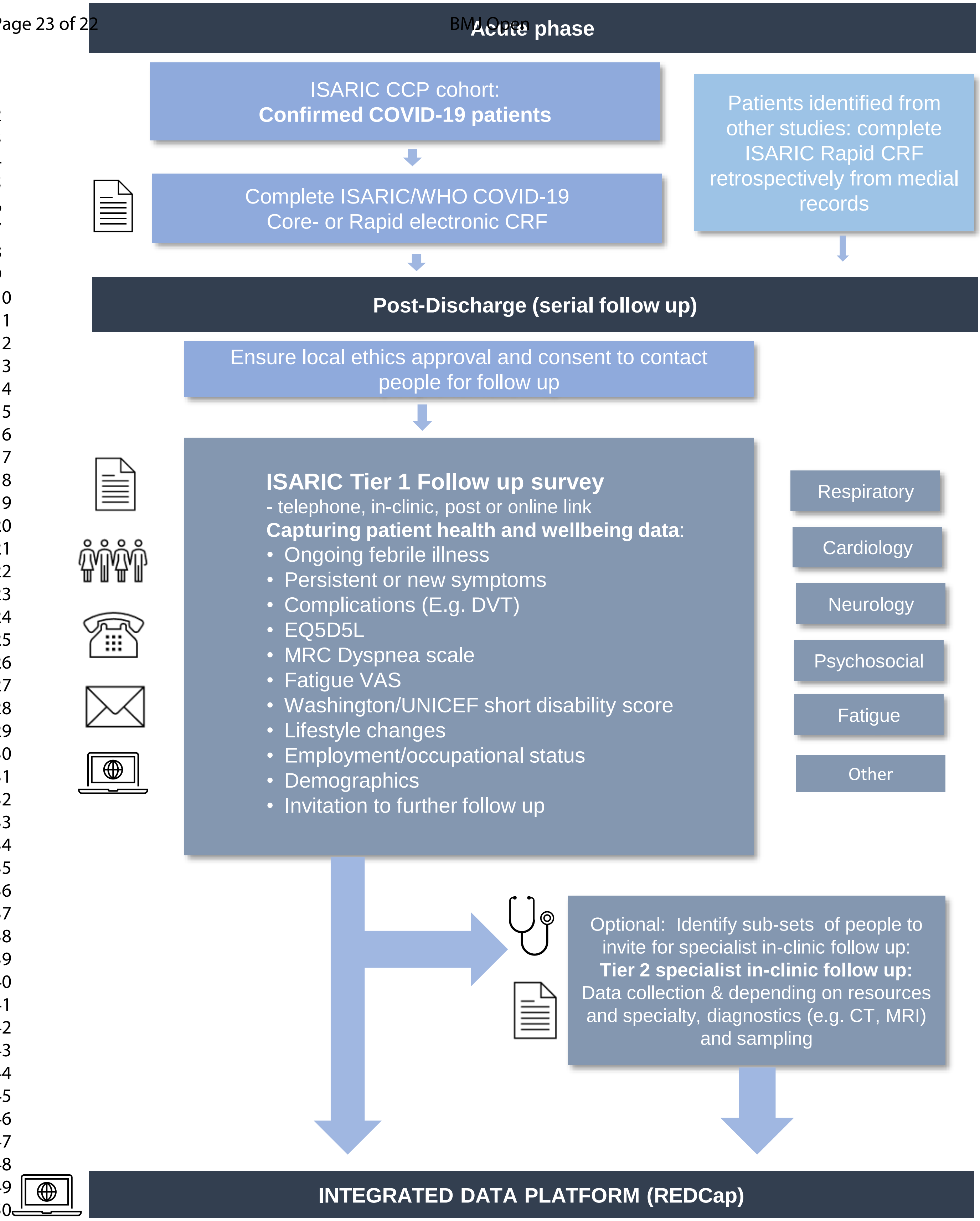
Acute phase
ISARIC CCP cohort:
Confirmed COVID-19 patients
Complete ISARIC/WHO COVID-19 Core- or Rapid electronic CRF
Patients identified from other studies: complete ISARIC Rapid CRF retrospectively from medial records
Post-Discharge (serial follow up)
Ensure local ethics approval and consent to contact people for follow up
ISARIC Tier 1 Follow up survey
- telephone, in-clinic, post or online link
Capturing patient health and wellbeing data:
• Ongoing febrile illness
• Persistent or new symptoms
• Complications (E.g. DVT)
• EQ5D5L
• MRC Dyspnea scale
• Fatigue VAS
• Washington/UNICEF short disability score
• Lifestyle changes
• Employment/occupational status
• Demographics
• Invitation to further follow up
Respiratory
Cardiology
Neurology
Psychosocial
Fatigue
Other
Optional: Identify sub-sets of people to invite for specialist in-clinic follow up:
Tier 2 specialist in-clinic follow up:
Data collection & depending on resources and specialty, diagnostics (e.g. CT, MRI) and sampling
INTEGRATED DATA PLATFORM (REDCap)

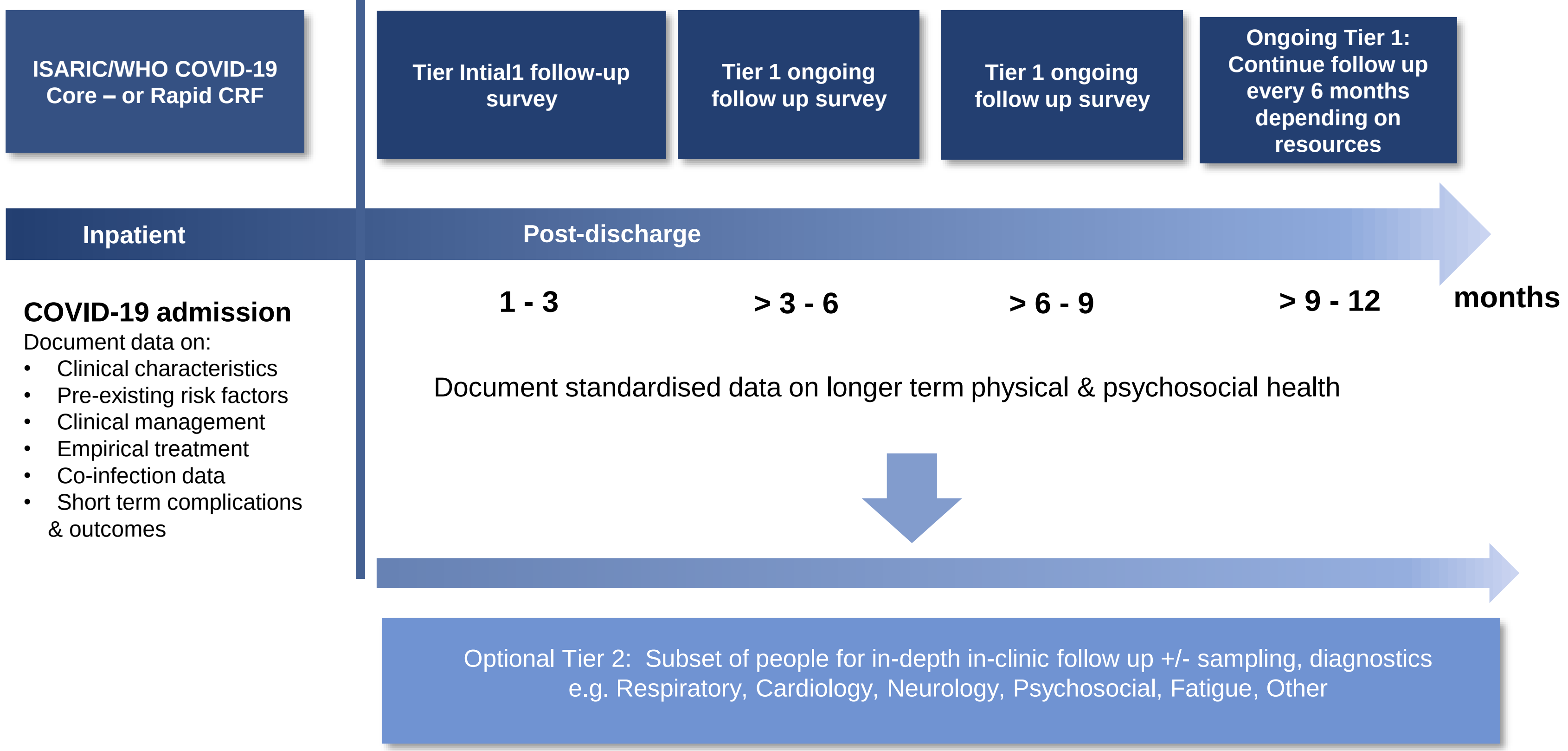
ISARIC/WHO COVID-19 Core – or Rapid CRF
Tier Intial1 follow-up survey
Tier 1 ongoing follow up survey
Tier 1 ongoing follow up survey
Ongoing Tier 1: Continue follow up every 6 months depending on resources
Inpatient
Post-discharge
1 - 3
> 3 - 6
> 6 - 9
> 9 - 12
months
COVID-19 admission
Document data on:
• Clinical characteristics
• Pre-existing risk factors
• Clinical management
• Empirical treatment
• Co-infection data
• Short term complications & outcomes
Document standardised data on longer term physical & psychosocial health
Optional Tier 2: Subset of people for in-depth in-clinic follow up +/- sampling, diagnostics
e.g. Respiratory, Cardiology, Neurology, Psychosocial, Fatigue, Other
